# scChronos: hierarchical temporal context learning for missing snapshot prediction in single-cell time series

**DOI:** 10.1093/bioinformatics/btag772

**Published:** 2026-10-14

**Authors:** Jiongsen Yao, Yunfei Zhang, Yong Xu, Jinjin Ma, Si Wu

**Affiliations:** School of Computer Science and Engineering, South China University of Technology, Guangzhou 510006, China; School of Future Technology, South China University of Technology, Guangzhou 510641, China; School of Computer Science and Engineering, South China University of Technology, Guangzhou 510006, China; The Institute of Future Health, South China University of Technology, Guangzhou 511442, China; School of Computer Science and Engineering, South China University of Technology, Guangzhou 510006, China

## Abstract

**Motivation:**

Single-cell time-series atlases provide discrete cross-sectional snapshots of continuous developmental processes, making missing-snapshot prediction essential for reconstructing trajectories and dynamic programs. However, destructive scRNA-seq profiling prevents tracking the same cells across time, yielding unpaired population snapshots and making missing-snapshot prediction challenging. Existing methods typically rely on explicit dynamical propagation or transport-based coupling between snapshots, which may ignore the statistical constraints of the overall population distribution and be sensitive to sampling noise and cell-to-cell variability.

**Results:**

We introduce scChronos, a hierarchical temporal context learning framework that formulates missing-snapshot prediction as target-conditioned population distribution prediction rather than individual-cell propagation. Unlike cell-centric propagation approaches, scChronos explicitly captures dual-level dynamics: (i) within each observed snapshot, scChronos compresses cell populations into cluster-representative cell representations with a transcriptomic encoder, preserving population information while reducing noise and (ii) across reference snapshots, the dual-branch temporal context integration module fuses information from all available and nearby time points into a temporal representation, which is then decoded with learnable cell-generation seeds to predict the target distribution. Extensive experiments demonstrate that scChronos improves both interpolation-style recovery and extrapolation-style forecasting compared with state-of-the-art methods. Beyond reducing distributional discrepancy, scChronos better preserves developmental topology and maintains cell-type-specific marker and pathway programs.

**Availability:**

The code and data are available at https://github.com/yjs193/scChronos and https://doi.org/10.5281/zenodo.20933283.

## 1 Introduction

The development of single-cell RNA sequencing (scRNA-seq) has revolutionized our ability to characterize cellular heterogeneity, identify cell states, and map gene-expression programs (Gao *et al.* 2024, Xue *et al.* 2026, Yao *et al.* 2026). While early studies largely focused on static snapshots of cell populations, recent advances have enabled time-series scRNA-seq experiments that capture cellular compositions and transcriptional states during development, differentiation, and disease progression (Schiebinger *et al.* 2019, Wei *et al.* 2025). Such temporal snapshots are essential for reconstructing dynamic cellular processes, yet the number of collected time points is often limited due to experimental cost and technical constraints (Trapnell *et al.* 2014). Predicting unobserved temporal snapshots from available time-point measurements therefore remains a critical and unmet need in temporal cell atlas construction (Sha *et al.* 2024, Shen *et al.* 2026).

Predicting missing single-cell snapshots is challenging due to several distinctive properties of temporal scRNA-seq data. First, scRNA-seq is inherently destructive. Each cell is measured once, eliminating any direct correspondence between cells at different time points (Weinreb *et al.* 2020). Second, temporal scRNA-seq datasets are typically sparse, containing only a limited number of measured time points with missing intermediate or future states (La Manno *et al.* 2018). Third, each observed snapshot comprises a distribution of heterogeneous cells undergoing continuous developmental programs (Saelens *et al.* 2019). Together, the above properties imply that predicting a missing snapshot requires not only modeling how individual cells transition over time but also constraining the predicted population distribution to conform to the statistical structure implicitly encoded across snapshots.

Traditional computational approaches have provided foundational tools for analyzing cellular dynamics from single-cell data. Pseudotime and trajectory-inference methods, such as Diffusion Pseudotime and Slingshot, order cells along developmental progressions and infer branching structures from transcriptomic similarity (Haghverdi *et al.* 2016, Street *et al.* 2018). RNA velocity and its extensions estimate short-term directional trends from spliced and unspliced RNA dynamics, enabling local prediction of cellular transitions (Bergen *et al.* 2020). While trajectory-inference and RNA-velocity methods have facilitated the study of developmental processes, they are primarily designed for inferring ordering, directionality, or trajectory structure within already observed cell populations. Predicting the transcriptomic distribution of an unobserved time point from reference snapshots is a fundamentally different task that the formulations are not designed to address.

Recent deep learning methods have been developed specifically for temporal single-cell prediction. Current approaches can be broadly grouped into three paradigms. Physical and dynamical system modeling methods, including PRESCIENT (Yeo *et al.* 2021) and PI-SDE (Jiang and Wan 2024), formulate cell differentiation as transitions along energy landscapes or as solutions to physics-informed stochastic differential equations. Neural ODE and optimal transport methods, such as MIOFlow (Huguet *et al.* 2022) and scNODE (Zhang *et al.* 2024), learn continuous-time dynamics via neural ordinary differential equations, often coupled with optimal transport. Sequence modeling approaches, exemplified by scMix (Han and Kim 2026), treat temporal prediction as a sequence prediction problem over multiple historical observations. Despite notable progress, existing deep learning methods share a fundamental limitation: prediction is formulated as propagating individual cell states across time, without explicitly constraining the predicted population distribution to match the statistical structure observed in reference snapshots. As a result, predictions from existing methods are sensitive to sampling noise and cell-to-cell variability. Furthermore, temporal information is typically integrated through local transitions or fixed historical windows, and current frameworks lack an adaptive mechanism to learn the relevance of each reference snapshot according to the target prediction context.

In this work, we formulate missing-snapshot prediction as target-conditioned population distribution generation rather than individual-cell propagation. Based on this formulation, scChronos is driven by two principles (Fig. 1). First, because propagating individual cell states is sensitive to technical noise and stochastic fluctuations, scChronos introduces within-snapshot aggregation: it compresses each observed snapshot into compact cluster-representative cell embeddings, filtering out cell-level noise while preserving population information. Second, to model developmental dynamics operating at multiple timescales, scChronos employs a dual-branch temporal context integration module, in which a global branch attends to all reference snapshots according to their relevance to the target time and a local branch captures immediate transitions from nearby time points. This design yields a fused temporal context representation, which is then combined with learnable generation seeds and decoded into the predicted target snapshot. Experimental results demonstrate that scChronos outperforms state-of-the-art baselines in both the recovery and forecasting tasks, achieving a relative reduction of over 8.2% in terms of mean OT distance compared with the second-best baseline, while faithfully preserving developmental manifolds, cell-type-specific marker programs, and pathway activities.

**Figure 1:**
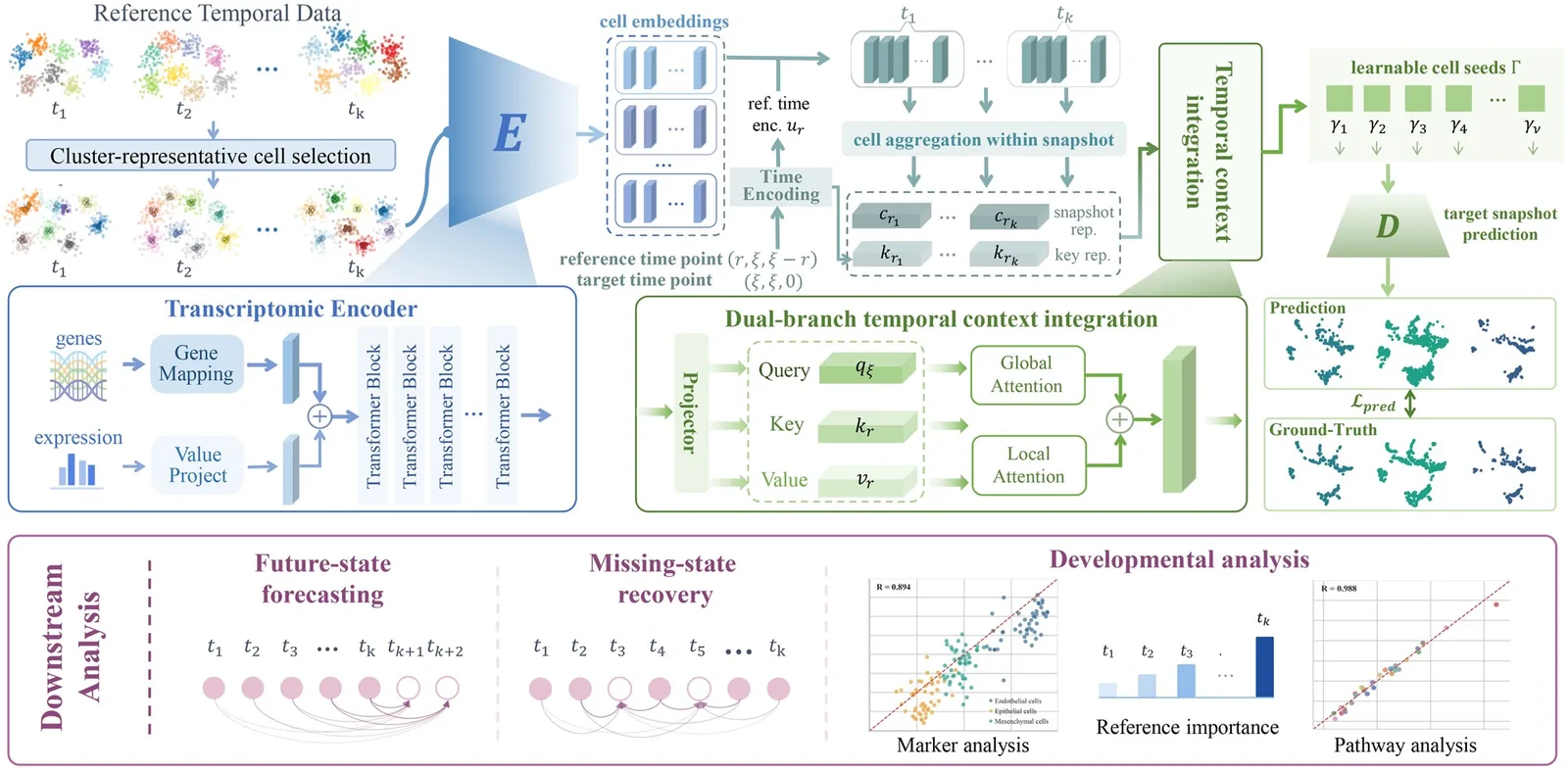
Overview of scChronos. scChronos predicts missing snapshots through a hierarchical temporal context learning framework. For each reference time point, cluster-representative cells are selected and encoded by a transcriptomic transformer, then aggregated into a compact representation to preserve population information. Given a target time point, scChronos integrates reference snapshots through a dual-branch temporal context integration module. The resulting representation is combined with learnable cell seeds, which act as target-cell output slots, and decoded into the predicted target snapshot distribution.

## 2 Materials and methods

### 2.1 Problem formulation

Let S⊂R denote the developmental time points. At each time point *s*, we observe a single-cell gene expression distribution $X_{s}={\left\{x_{i}^{\left(s\right)}\in R^{G}\right\}}_{i=1}^{N_{s}}$, where *G* is the number of genes and $N_{s}$ is the number of cells. Given the target time point ξ, let $C_{\xi }\subset S\setminus \{\xi \}$ denote the available reference time points. In missing-state recovery, $C_{\xi }$ may include observations on either side of the held-out target, whereas in future-state forecasting all held-out targets use the same fixed set of observed historical snapshots preceding the forecasting horizon. scChronos predicts the target snapshot by learning a mapping from the available observed snapshots and the target time:


(1) $${\hat{X}}_{\xi }=f_{\Theta }\left({\left\{(X_{r},r)\right\}}_{r\in C_{\xi }},\xi \right),$$

where $f_{\Theta }$ denotes scChronos with learnable parameters Θ, ${\hat{X}}_{\xi }\in R^{\nu \times G}$ is the predicted target snapshot with ν generated target cells. The learning objective is to predict a target cell population ${\hat{X}}_{\xi }$ that approximates the ground-truth snapshot $X_{\xi }$.

### 2.2 Cluster-representative cell transcriptomic encoding

To obtain a compact yet representative description of each reference snapshot, for each reference time point $r\in C_{\xi }$, scChronos first selects cluster-representative cells that summarize the major population information of the reference snapshot $X_{r}$. Specifically, PCA was fitted on the available reference cells within each split, and KMeans was then applied to the PCA embeddings of cells from each reference time point. Cells nearest to the cluster centroids were retained to form the cluster-representative cell set $P_{r}$. Details of the selection procedure are provided in Section 5, available as supplementary data at *Bioinformatics* online.

For a cluster-representative cell $p\in P_{r}$ with the expressed-gene set $G_{p}$, gene mapping and value projection represent the expressed genes and their nonzero expression values as gene-identity embeddings $e_{p}^{\mathrm{gene}}$ and expression-value embeddings $e_{p}^{\mathrm{expr}}$. Gene-identity embeddings specify which genes are observed in the cluster-representative cell, whereas expression-value embeddings encode their measured abundance. The cluster-representative cell embedding is then obtained by:


(2) $$h_{p}=E(e_{p}^{\mathrm{expr}},e_{p}^{\mathrm{gene}}),$$

where *E* is the trainable cell encoder. To strengthen gene-level transcriptomic information in the compact cluster-representative cell embeddings, we introduce a masked cell reconstruction objective. For each cluster-representative cell *p*, a subset of expressed genes $M_{p}\subset G_{p}$ is randomly masked. The encoder maps the masked token sequence $Q_{p}$ to a masked cluster-representative cell embedding ${\tilde{h}}_{p}=E(Q_{p})$, and a reconstruction head predicts the masked expression values as $\hat{p}=D_{p}({\tilde{h}}_{p})$. The masked reconstruction loss is:


(3) $$L_{\text{cell}}=E_{p,M_{p}}\left[\frac{1}{\left|M_{p}\right|}\sum_{g\in M_{p}}{\left({\hat{p}}_{g}-p_{g}\right)}^{2}\right],$$

where $p_{g}$ and ${\hat{p}}_{g}$ denote the original and reconstructed expression values of gene *g*, respectively, and $M_{p}$ denotes the masked expressed-gene subset. Minimizing $L_{\text{cell}}$ encourages the encoder to recover masked expression values from the remaining genes, preserving gene-level information in cluster-representative cell embeddings.

### 2.3 Cluster-representative-cell-to-snapshot temporal aggregation

To summarize each reference snapshot, scChronos weights and combines cluster-representative cell embeddings according to their relevance to the target time point. Given a target time point ξ and a reference time point *r*, the temporal encoding is:


(4) $$u_{r}=\psi (r,\xi ,\xi -r),$$

where ψ(·) denotes a learnable time-encoding function. Given the cluster-representative cell embeddings ${\left\{h_{m}\right\}}_{m=1}^{M}$ associated with $P_{r}$ from reference snapshot *r*, a learnable relevance function *F* computes target-conditioned scores for the cluster-representative cells:


(5) $$\alpha_{m}=\sigma_{m}\left(F(h_{m},u_{r})\right),$$

where $\sigma_{m}(\cdot )$ denotes softmax normalization over cells in reference snapshot *r*, and $\alpha_{m}$ denotes the normalized relevance weight of cluster-representative cell *m*. The reference snapshot representation is then obtained by:


(6) $$c_{r}=\sum_{m=1}^{M}\alpha_{m}h_{m},$$

where $c_{r}$ denotes the target-conditioned representation of reference snapshot *r*. To retain snapshot-level distributional information, scChronos further aligns summary statistics decoded from $c_{r}$ with those computed from the selected cluster-representative cells, using an MLP O(·) to predict the gene-wise mean and standard deviation from $c_{r}$:


(7) $$({\hat{\mu }}_{r},{\hat{\sigma }}_{r})=O(c_{r}),$$

which are compared with the empirical gene-wise mean $\mu_{r}$ and standard deviation $\sigma_{r}$ of the cluster-representative cell set:


(8) $$L_{\text{snap}}=E_{r,\xi }\left[\|{\hat{\mu }}_{r}-\mu_{r}{\|}_{2}^{2}+\|{\hat{\sigma }}_{r}-\sigma_{r}{\|}_{2}^{2}\right],$$

where $\|\cdot {\|}_{2}$ denotes the Euclidean norm. The loss aligns statistics decoded from $c_{r}$ with empirical statistics of the selected cluster-representative cells, further encouraging the snapshot representation to preserve gene-wise distributional statistics.

### 2.4 Dual-branch temporal context integration

Temporal prediction requires integrating information from multiple reference snapshots, where nearby snapshots often provide direct transition cues and distant snapshots may contain broader developmental context. To capture both types of temporal information, scChronos uses a dual-branch temporal context integration module, leveraging the snapshot representations ${\left\{c_{r}\right\}}_{r\in C_{\xi }}$. For the target time point ξ, the target-time encoding is defined as $u_{\xi }=\psi (\xi ,\xi ,0)$, and the target query is computed as $q_{\xi }=g_{Q}(u_{\xi })$. For each reference time point *r*, the key and value embeddings are computed as:


(9) $$k_{r}=g_{K}(c_{r},u_{r}),$$

(10) $$v_{r}=g_{V}(c_{r}),$$

where $g_{Q}$, $g_{K}$, and $g_{V}$ are learnable projections. The query embedding $q_{\xi }$ encodes the target time point, the key embedding $k_{r}$ measures the relevance of reference snapshot *r* to the target time, and the value embedding $v_{r}$ carries the reference snapshot information to be aggregated. The global branch performs cross-attention over all reference snapshots:


(11) $$\beta_{r}=\frac{\;\text{exp}(q_{\xi }^{⊤}k_{r})}{\sum_{r^{\prime }\in C_{\xi }}\;\text{exp}\;(q_{\xi }^{⊤}k_{r^{\prime }})},$$

(12) $$z_{\xi }^{G}=\sum_{r\in C_{\xi }}\beta_{r}v_{r},$$

where $\beta_{r}$ denotes the normalized reference-snapshot weight across all $r\in C_{\xi }$. In parallel, a local branch applies cross-attention to the κ temporally closest reference snapshots. Specifically, $N_{\kappa }(\xi )$ contains the κ reference time points in $C_{\xi }$ with the smallest absolute temporal distances |r−ξ|. In missing-state recovery, these references may occur either before or after the target time point, whereas in future-state forecasting they are restricted to preceding observations. The local branch then aggregates $N_{\kappa }(\xi )$ to produce a local context embedding $z_{\xi }^{L}$. In our experiments, we set κ=2, as supported by parameter analysis in Fig. 7, available as supplementary data at *Bioinformatics* online. The final temporal context representation is obtained by:


(13) $$z_{\xi }=z_{\xi }^{G}+z_{\xi }^{L},$$

which integrates within-snapshot information with across-snapshot temporal information. Given the fused temporal context $z_{\xi }$, scChronos predicts the target snapshot with a gene expression decoder. Let $\Gamma ={\left\{\gamma_{j}\right\}}_{j=1}^{\nu }$ denote ν learnable cell-generation seeds, where each seed $\gamma_{j}$ serves as a target-cell output slot, allowing the decoder to generate an unordered set of target cells conditioned on the same temporal context. The predicted target snapshot is obtained by:


(14) $${\hat{X}}_{\xi }=D_{\mathrm{pred}}(z_{\xi },\Gamma ),$$

where $D_{\mathrm{pred}}$ is the gene expression predictor, and ${\hat{X}}_{\xi }\in R^{\nu \times G}$ denotes the predicted target expression matrix. The seed set Γ is shared across target time points, while target specificity is introduced through $z_{\xi }$. We set ν=128 per decoding pass and repeat decoding as needed during evaluation to match the target population size.

### 2.5 Model optimization

The primary training objective compares the predicted and ground-truth target cell populations using the Wasserstein distance, approximated with debiased Sinkhorn divergence (Cuturi 2013, Peyré and Cuturi 2019):


(15) $$L_{\text{pred}}=E_{\xi }\left[W_{OT}\left({\hat{X}}_{\xi },X_{\xi }\right)\right],$$

where $W_{OT}(\cdot ,\cdot )$ denotes the Wasserstein distance.

Together with the auxiliary cell-level $L_{\text{cell}}$ loss and snapshot-level loss $L_{\text{snap}}$, the overall objective is:


(16) $$\underset{\Theta }{\min}\;L_{\text{pred}}+\lambda_{\text{cell}}L_{\text{cell}}+\lambda_{\text{snap}}L_{\text{snap}},$$

where Θ denotes all learnable parameters in scChronos, and $\lambda_{\text{cell}}$ and $\lambda_{\text{snap}}$ control the contribution of the auxiliary losses. In our experiments, we set $\lambda_{\text{cell}}=0.05$, $\lambda_{\text{snap}}=0.5$, as supported by the parameter analysis in Fig. 8, available as supplementary data at *Bioinformatics* online. The joint objective enables scChronos to predict target cell distribution while preserving both cell-level and snapshot-level information.

## 3 Results

### 3.1 Datasets and implementation

To evaluate scChronos for missing-snapshot prediction, we collected four temporal scRNA-seq datasets spanning human and mouse developmental systems. The human datasets include Veres and Cao 2020 (Veres *et al.* 2019, Cao *et al.* 2020), and the mouse datasets include Schiebinger2019 and ECED Kidney (Schiebinger *et al.* 2019, Lin *et al.* 2026). All datasets were preprocessed following the standard pipeline used in scMix: raw count matrices were normalized, log-transformed, and restricted to highly variable genes (Wolf *et al.* 2018). Dataset descriptions and task splits are provided in Tables 1 and 2, available as supplementary data at *Bioinformatics* online. For each dataset-task-HVG setting, all methods used the same train-target split, target time points, and HVG panel and were evaluated against the same held-out target snapshots using the same OT-distance evaluation procedure. Definitions of metrics are provided in Section 2, available as supplementary data at *Bioinformatics* online, and more implementation details are provided in Section 3, available as supplementary data at *Bioinformatics* online.

### 3.2 scChronos improves snapshot prediction performance

We first evaluated the predictive performance of scChronos on the missing snapshot prediction task. Specifically, we compared scChronos against five state-of-the-art temporal prediction methods across the four datasets for missing-state recovery and future-state forecasting. For each target time point, predicted and ground-truth cell populations were compared using the optimal transport (OT) distance across five independent runs with different random seeds (Peyré and Cuturi, 2019). Figure 2 summarizes the benchmark results. More detailed results are provided in Tables 5–28, available as supplementary data at *Bioinformatics* online.

**Figure 2:**
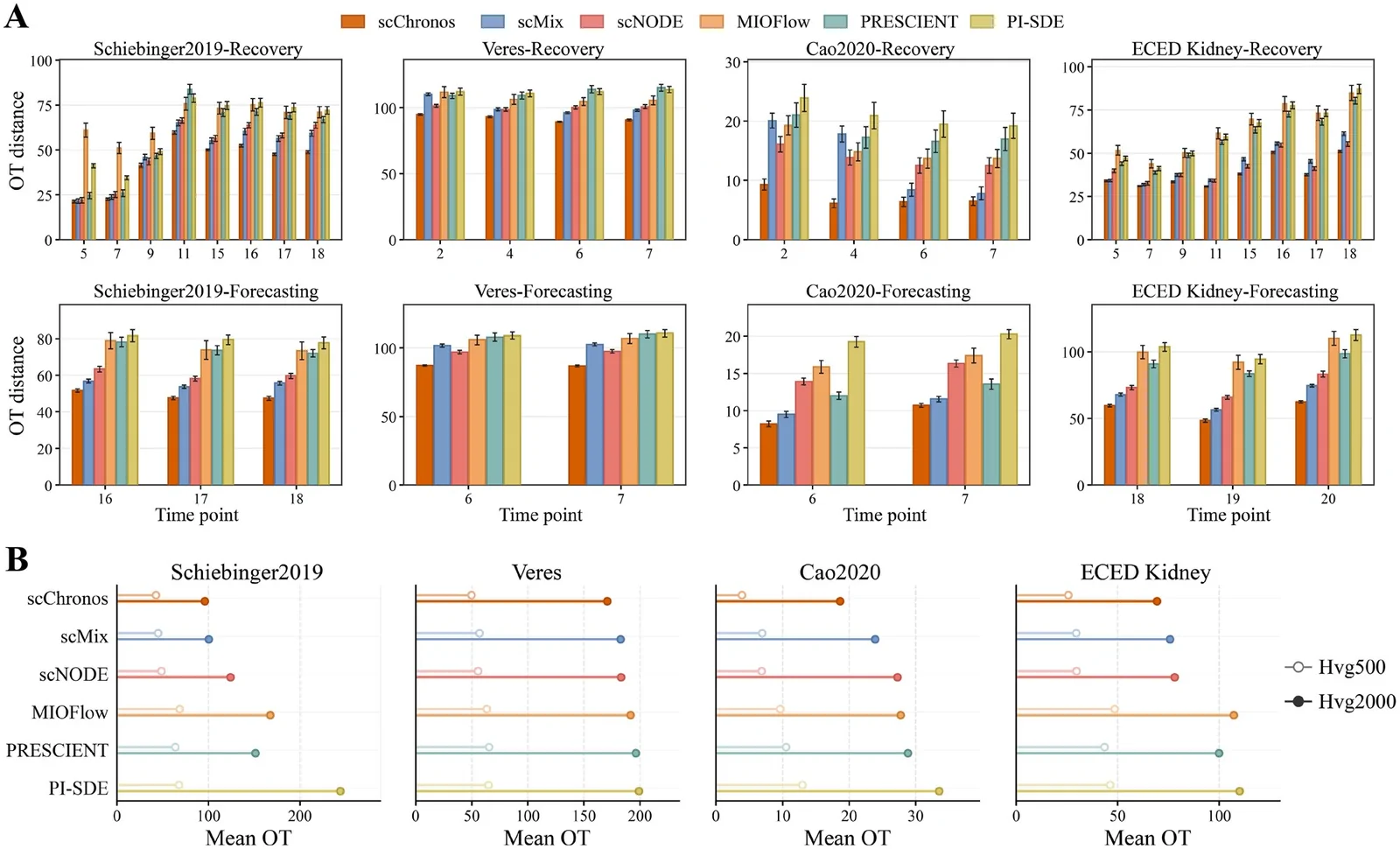
(A) Per-target-time OT distances on HVG1000 recovery and forecasting tasks across four datasets. (B) Average OT distances under HVG500 and HVG2000 settings, showing that scChronos maintains robust performance across different feature scales.

On the datasets with 1000 highly variable genes (HVG1000), scChronos achieved the lowest mean OT distance in all eight dataset-task combinations (Fig. 2A). The improvement was consistent in both the recovery and forecasting tasks. Compared with scMix, scChronos reduced the mean OT distance from 43.37 to 38.32 on ECED Kidney recovery and from 55.44 to 48.89 on Schiebinger2019 forecasting. We further evaluated the robustness of scChronos across different numbers of highly variable genes. Under both HVG500 and HVG2000 settings, scChronos retained the best overall performance (Fig. 2B).

To evaluate whether predicted cells preserve the manifold of the observed developmental landscape, we visualized predicted target cells using UMAP (McInnes *et al.* 2018). Specifically, all predictions were projected onto a shared UMAP coordinate system fitted on the ground-truth cell populations, allowing predicted cells to be compared directly. Figure 3 shows representative recovery results on Schiebinger2019 and ECED Kidney. As shown in Fig. 3A and B, scChronos predictions were distributed along the major ground-truth manifold, whereas several baseline methods showed off-manifold shifts or overly concentrated predictions at some target time points. Figure 3C and D provide per-time-point comparisons between scChronos predictions and the corresponding ground-truth snapshots. Across both datasets, predicted cells aligned well with the target-specific ground-truth regions, suggesting that the OT improvement reflects better manifold fidelity rather than only aggregate expression matching. Additional results are provided in Figs 1–6, available as supplementary data at *Bioinformatics* online.

**Figure 3:**
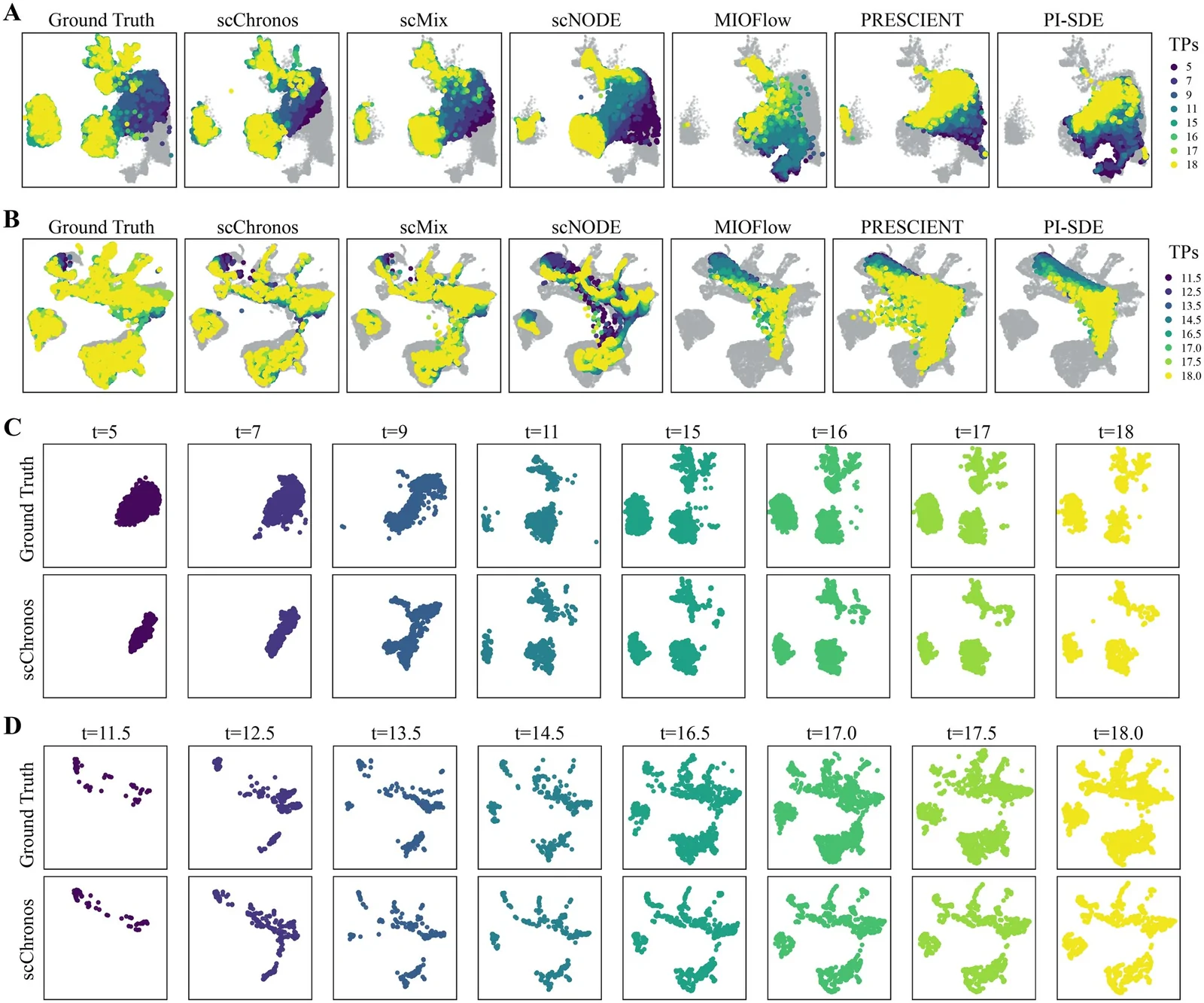
Manifold-level evaluation of recovery predictions. (A) UMAP visualization of Schiebinger 2019 HVG1000 recovery predictions from scChronos and baseline methods. Gray points denote reference cells, and colored points denote ground-truth or predicted target cells. (B) UMAP visualization of ECED Kidney HVG1000 recovery predictions under the same setting. (C) Per-target-time comparison between Schiebinger 2019 ground truth and scChronos predictions. (D) Per-target-time comparison between ECED Kidney ground truth and scChronos predictions.

**Figure 4:**
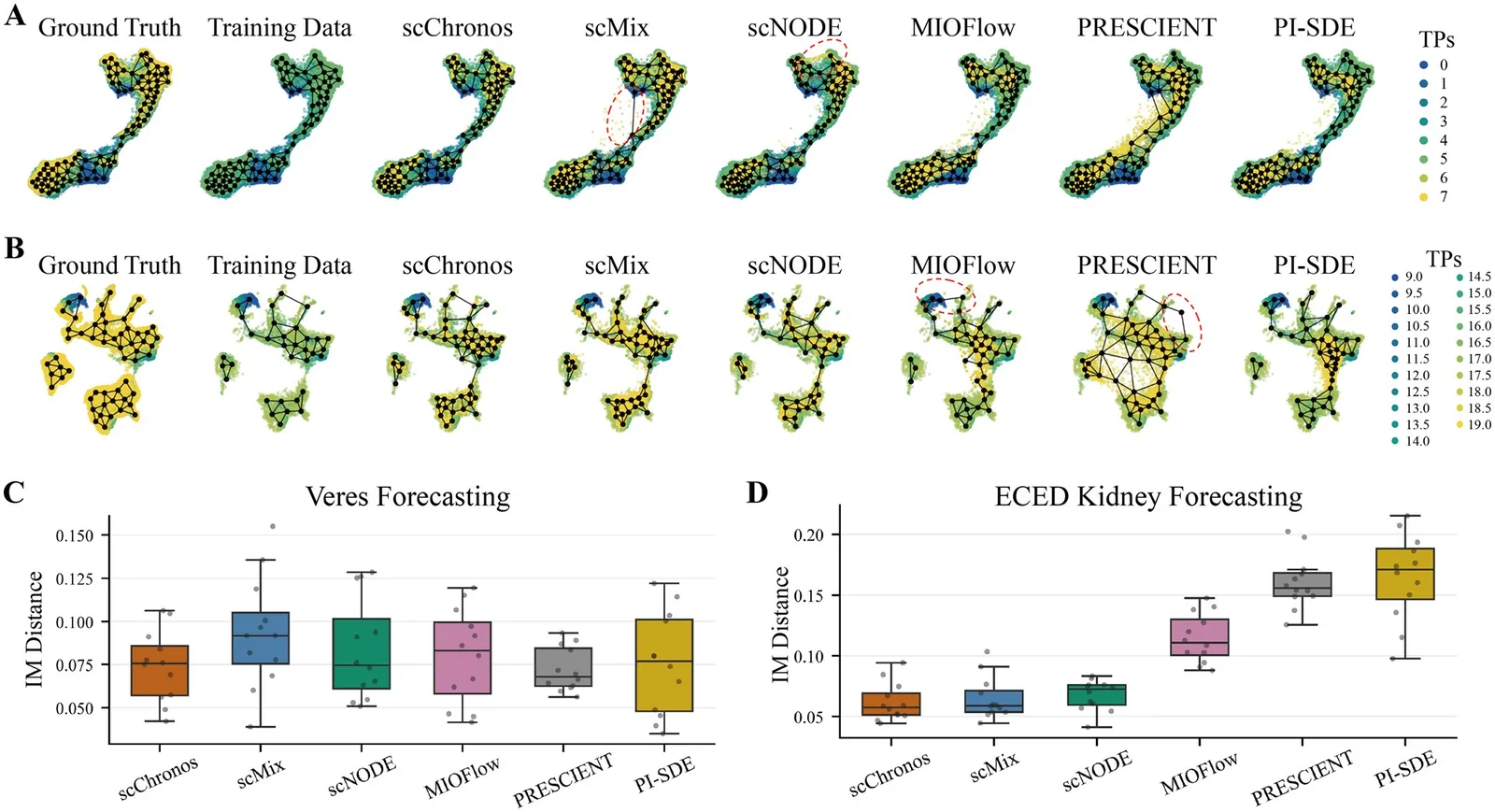
Trajectory-topology evaluation in the forecasting task. (A) PAGA-like trajectory visualization on Veres HVG1000 dataset, where nodes represent cluster centers and edges represent neighborhood connections between clusters in the UMAP space. (B) PAGA-like trajectory visualization on ECED Kidney HVG1000. (C) Ipsen–Mikhailov distance between predicted and ground-truth target-cell kNN graphs for Veres. (D) Ipsen–Mikhailov distance between predicted and ground-truth target-cell kNN graphs for ECED kidney. Lower values indicate closer graph topology.

**Figure 5:**
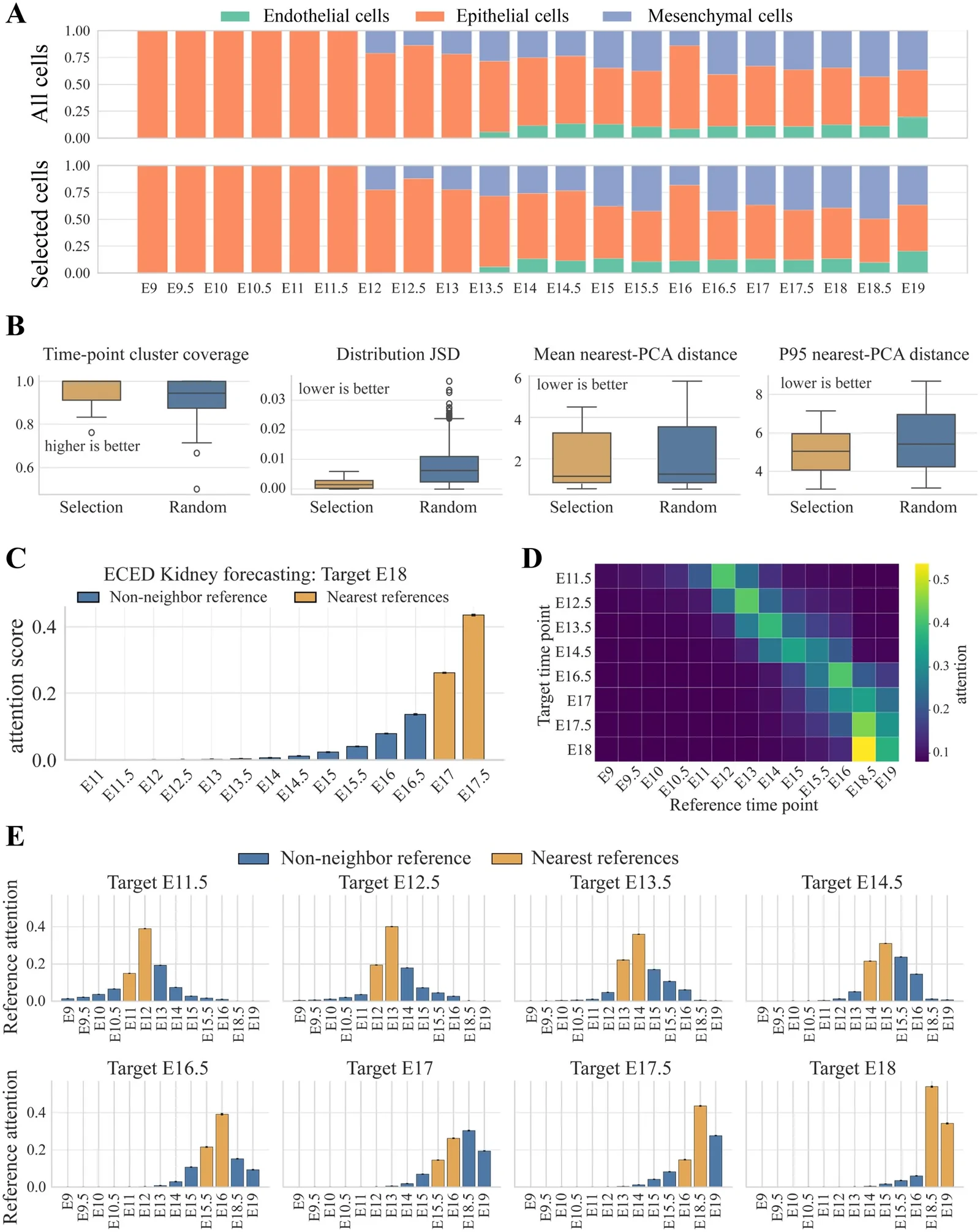
(A) Cell-type composition of all ECED kidney cells and selected cluster-representative cells. (B) Quantitative comparison between cluster-representative cells and randomly sampled cells. (C) Reference-attention profile for ECED kidney forecasting at target E18. (D) Reference-attention heatmap across target and reference time points in ECED kidney recovery. (E) Per-target recovery attention profiles in ECED kidney recovery. The attention values in (C–E) correspond to the global-branch weights, where larger values indicate greater weighting of the corresponding reference snapshot.

**Figure 6:**
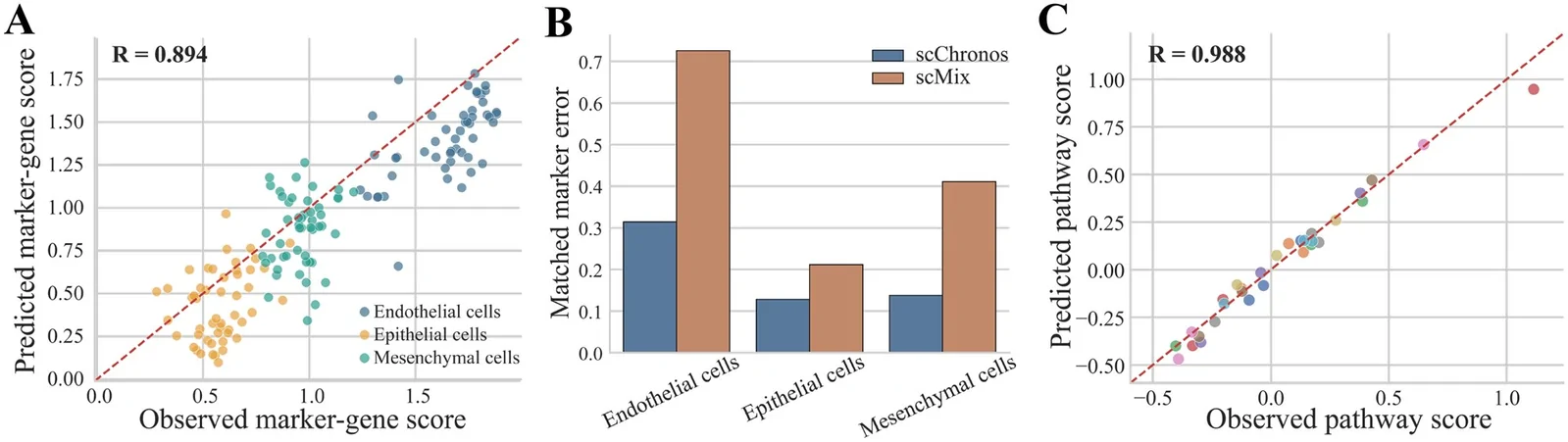
Biological program preservation in ECED Kidney future-state forecasting. (A) Predicted versus ground-truth marker-gene scores for endothelial, epithelial, and mesenchymal cells at E19. (B) Matched marker-program error for scChronos and scMix across cell types. (C) Predicted versus ground-truth pathway scores for E19 target cells, computed from predicted and ground-truth gene expression profiles using ssGSEA. The displayed pathways are related to developmental signaling, and Pearson correlation was computed across the displayed cell-type-by-pathway scores.

### 3.3 scChronos achieves greater trajectory preservation

To further evaluate whether the predicted snapshots maintain coherent developmental trajectories, we constructed PAGA-like graphs based on predicted target cells together with reference cells (Wolf *et al.* 2019). Here, “PAGA-like” refers to a cluster-level graph abstraction of the UMAP manifold, distinct from the target-cell kNN graphs used for IM-distance calculation. In the Training Data Only panels, target time points are absent, leading to incomplete graph structures. A reliable prediction method should recover a graph topology that closely approximates the ground truth without introducing spurious connections. As shown in Fig. 4A, scChronos reconnected the future branch and produced a graph structure similar to the observed trajectory in the Veres forecasting task. By contrast, scMix showed a spurious connection, and scNODE produced a mismatched region. As shown in Fig. 4B, in the ECED Kidney forecasting task, scChronos also recovered the major late target regions and maintained the main graph organization. MIOFlow failed to recover part of the upper branch, whereas PRESCIENT distorted the local topology. To quantify the graph-level differences, we further computed the Ipsen–Mikhailov (IM) distance between predicted and ground-truth target-cell kNN graphs (Ipsen and Mikhailov 2002). scChronos achieved a mean IM distance of 0.0740 in Veres forecasting, compared with 0.0930 for scMix, and obtained the lowest mean IM distance in ECED Kidney forecasting, with 0.0616 versus 0.068 for scNODE (Fig. 4C and D). Together, the PAGA visualizations and IM distance quantifications demonstrate that scChronos better preserves trajectory organization in future-state prediction, consistent with its superior performance in snapshot-level recovery and forecasting.

### 3.4 scChronos learns hierarchical temporal context for target-adaptive prediction

To characterize temporal reference context learning in scChronos, we first evaluated whether cluster-representative cells approximate each reference snapshot at the population level. As shown in Fig. 5A, selected cluster-representative cells closely preserved endothelial, epithelial, and mesenchymal cell-type proportions across developmental time points, maintaining the coarse cellular composition of the original snapshots. Moreover, compared with random sampling, cluster-representative cells provided higher time-point cluster coverage, lower distribution JSD and mean nearest-PCA distance (Fig. 5B). The results suggest that cluster-representative cells serve as compact cellular anchors for reference snapshots.

To understand how scChronos integrates multi-time-point information for a given target time point, we analyzed the learned attention weights across reference snapshots. For E18 forecasting, scChronos assigned larger attention to late reference time points, especially E17 and E17.5 (Fig. 5C), which are temporally close to the target and occupy regions similar to late target snapshots in the ground-truth UMAP visualization (Fig. 1, available as supplementary data at *Bioinformatics* online). In the recovery task, reference attention varied across target time points and combined nearest references with non-neighbor references when broader temporal context was informative (Fig. 5D and E). Together, the cluster-representative cell and attention analyses show that scChronos builds target-conditioned temporal context by selecting cluster-representative cells that preserve snapshot-level population information and adaptively weighting reference time points according to target-snapshot relevance. Ablation analyses of cluster-representative cell selection and the local/global fusion branches are provided in Table 4, available as supplementary data at *Bioinformatics* online.

### 3.5 scChronos preserves cell-type marker programs and pathway activities

To evaluate whether predicted target snapshots retain interpretable biological programs, we analyzed marker-gene and pathway activities in ECED Kidney E19 forecasting. As shown in Fig. 6A, scChronos predictions closely followed the ground-truth marker-gene scores across endothelial, epithelial, and mesenchymal cells, with a Pearson correlation of 0.894. Figure 6B shows that scChronos achieved lower matched marker-program error than scMix in all three terminal cell types, indicating more faithful preservation of cell-type-specific transcriptional programs. At the pathway level, we used ssGSEA with Reactome gene sets to compute pathway activity scores from both predicted and ground-truth gene expression profiles (Joshi-Tope *et al.* 2005, Fang *et al.* 2023). As shown in Fig. 6C, the pathway scores derived from predicted expression closely matched those derived from ground-truth expression, reaching a Pearson correlation of 0.988. Overall, the results suggest that scChronos preserves marker-level and pathway-level biological programs in prediction tasks, rather than merely reducing distributional discrepancy.

## 4 Discussion

scChronos presents a temporal context modeling framework for single-cell snapshot prediction. Instead of treating temporal prediction as the propagation of individual cell states across unpaired observations, scChronos models reference snapshots and predicts the target cell-state distribution from target-conditioned reference context. This formulation is aligned with the nature of time-series scRNA-seq data, where destructive profiling prevents cell-to-cell correspondence across time and each time point represents a population-level snapshot of an underlying developmental process. By integrating cluster-representative cell embedding, cell-to-snapshot aggregation, and a dual-branch temporal context integration module, scChronos captures population-level snapshot information and target-relevant temporal context, thereby improving the prediction of missing single-cell snapshots.

Extensive experimental results demonstrate that scChronos improves temporal prediction performance. Across four datasets, two prediction settings and multiple HVG scales, scChronos achieved lower distributional discrepancy than competing methods. The UMAP and PAGA analyses show that the predicted cells better preserve developmental manifold geometry and trajectory topology. The marker-gene and pathway analyses further show that forecasted target snapshots retain cell-type-specific transcriptional programs and pathway activities. The ablation analysis in Table 4, available as supplementary data at *Bioinformatics* online, further supports the design of scChronos. Removing cluster-representative cell selection or the global fusion branch caused the largest performance drops on ECED kidney recovery, whereas removing the local fusion branch led to a smaller but consistent increase in OT distance. The ablation results suggest that cluster-representative cell selection and the global fusion branch provide the main predictive gains, while the local fusion branch further helps maintain local temporal consistency.

Looking forward, scChronos could be extended toward finer-grained and more transferable temporal modeling. Although cluster-representative cells provide compact snapshot representations, rare or transient cell states may be underrepresented. In addition, scChronos predicts target-cell distributions rather than matched future identities. Given cellular heterogeneity and the complexity of developmental processes, incorporating additional biological constraints could further improve the generalization of temporal snapshot prediction.

## Supplementary Material

btag772_Supplementary_Data

## Data Availability

All single-cell RNA-seq datasets used in the present study are publicly available. The Schiebinger2019 dataset is available from the Waddington-OT resources. The Veres dataset is available from GEO (GSE114412). The Cao 2020 dataset is obtained from the CZ CELLxGENE collection *A human cell atlas of fetal gene expression*. The ECED Kidney dataset can be accessed through OMIX007369-13.
