# Supplementary material for: scChronos: hierarchical temporal context learning for missing snapshot prediction in single-cell time series
Source: Bioinformatics. 2026 Oct 14;42(10):btag772. doi: 10.1093/bioinformatics/btag772 (PMC13722282; doi:10.1093/bioinformatics/btag772)
Supplement: Supplementary file 1 [file btag772_supplementary_data.pdf]

Supplementary Material

S1 Dataset Description

We evaluated scChronos on four time-series single-cell transcriptomic datasets covering mouse and human developmental systems. For each dataset, we used the same processed split convention as the benchmark experiments. Gene expression matrices were represented as highly variable gene (HVG) panels and were normalized and log-transformed before model fitting and evaluation. Table S1 provides dataset descriptions.

Table S1: Datasets used in the benchmark.

Dataset	Species	# Cells	# Time points
Schiebinger2019	Mouse	23,619	19
ECED Kidney	Mouse	20,805	21
Veres2019	Human	51,274	8
Cao2020	Human	43,618	8

The two prediction tasks follow the same split logic across datasets. In the missing-state recovery task, one or more observed intermediate time points are held out and predicted from the remaining reference time points. In the future-state forecasting task, later time points are held out and predicted from earlier reference time points. Table S2 summarizes the target time points used for evaluation. For ECED Kidney, numerical indices correspond to ordered developmental stages, and the stage labels are shown in the UMAP visualizations.

Table S2: Evaluation splits used by the recovery and forecasting tasks.

Dataset	Task	Reference time points	Target time points
Schiebinger2019	Recovery	0, 1, 2, 3, 4, 6, 8, 10, 12, 13, 14	5, 7, 9, 11, 15, 16, 17, 18
	Forecasting	0, 1, 2, 3, 4, 5, 6, 7, 8, 9, 10, 11, 12, 13, 14, 15	16, 17, 18
ECED Kidney	Recovery	0, 1, 2, 3, 4, 6, 8, 10, 12, 13, 14, 19, 20	5, 7, 9, 11, 15, 16, 17, 18
	Forecasting	0, 1, 2, 3, 4, 5, 6, 7, 8, 9, 10, 11, 12, 13, 14, 15, 16, 17	18, 19, 20
Veres2019	Recovery	0, 1, 3, 5	2, 4, 6, 7
	Forecasting	0, 1, 2, 3, 4, 5	6, 7
Cao2020	Recovery	0, 1, 3, 5	2, 4, 6, 7
	Forecasting	0, 1, 2, 3, 4, 5	6, 7

S2 Definitions of Metrics

S2.1 Optimal Transport Distance

The primary quantitative metric is the optimal transport (OT) distance between the predicted and ground-truth target snapshots. For a target time point ξ, we first compute the pairwise Euclidean distance matrix between predicted cells and ground-truth cells:

$$d_{ij}^{(\xi)} = \|\widehat{x}_{\xi,i} - x_{\xi,j}\|_2.$$

We then computed a one-to-one matching between predicted and ground-truth cells using the Hungarian linear assignment algorithm on $d^{(\xi)}$:

$$\pi^* = \arg\min_{\pi} \sum_{(i,j)\in\pi} d_{ij}^{(\xi)}, \quad D_{\mathrm{OT}}(\widehat{\mathcal{X}}_\xi, \mathcal{X}_\xi) = \frac{1}{|\pi^*|} \sum_{(i,j)\in\pi^*} d_{ij}^{(\xi)}.$$

Lower OT distance indicates that the predicted target snapshot is closer to the ground-truth target snapshot. The dataset-level score is computed as the arithmetic mean of OT distances over all target time points.

S2.2 Ipsen–Mikhailov Graph Distance

For trajectory-level evaluation, we used the Ipsen–Mikhailov (IM) distance to compare graph topology between predicted and ground-truth target cells. For each target time point ξ, we constructed kNN graphs $\widehat{G}_\xi$ and G_ξ from the predicted snapshot $\widehat{\mathcal{X}}_\xi$ and the ground-truth snapshot $\mathcal{X}_\xi$, respectively, using the same graph construction parameters. The IM distance compares the smoothed Laplacian spectra of two graphs:

$$\mathrm{IM}(\widehat{G}_\xi, G_\xi) = \left\| \rho_{\widehat{G}_\xi} - \rho_{G_\xi} \right\|_2,$$

where $\rho_{\widehat{G}_\xi}$ and ρ_{G_ξ} denote the spectral densities derived from the graph Laplacian eigenvalues of the predicted and ground-truth graphs. Lower IM distance indicates more similar trajectory topology.

S2.3 Attention Score

For each target time point, scChronos assigns an attention score to every reference time point. For notation simplicity, the target-time subscript is omitted below. Given the target query q and the reference key k_r, the attention score for reference time point r is computed as

$$w_r = \mathrm{softmax}\left(q^\top k_r\right),$$

where the scores are normalized over all reference time points, and a larger w_r indicates that the corresponding reference snapshot contributes more to the target prediction.

S2.4 Marker Program Recovery

Marker gene sets were derived from the ground-truth target snapshot. For ECED Kidney E19 forecasting, cells were grouped by the annotated cell types in the original metadata, including endothelial, epithelial and mesenchymal cells. For each cell type, genes were ranked by cell-type specificity using differences in mean expression and detection frequency between that cell type and all remaining cells. We retained the top 50 marker genes for each cell type after restricting genes to the HVG1000 panel used by the prediction models.

Because predicted cells do not carry observed cell-type annotations, we assigned pseudo cell-type labels to predicted cells during evaluation. Specifically, a random forest classifier was trained using the ground-truth target-cell expression profiles and their metadata cell-type labels, and the trained classifier was then applied to the predicted expression matrix. The pseudo labels were used only for cell-type-aware evaluation and were not used as model input.

For each cell type, the marker program score was computed as the average expression of its marker genes within the corresponding cell group. Ground-truth scores were computed using metadata cell-type labels, whereas predicted scores were computed using classifier-assigned pseudo labels. Marker recovery was summarized by the predicted-versus-ground-truth correlation and the matched marker-program error.

S2.5 Pathway Program Recovery

Pathway activity scores were computed using ssGSEA with Reactome gene sets. Reactome mouse gene sets were obtained from MSigDB/Reactome and restricted to genes present in the HVG1000 panel. Pathways with fewer than five overlapping genes were removed. The ssGSEA scores were computed using the `gseapy` package with the same Reactome gene sets for predicted and ground-truth expression profiles.

S3 Implementation Details

All baseline models are obtained from their official repositories, ensuring reproducibility and consistency. All runs for scChronos used 256 cluster-representative cells, hidden dimension 256, weight decay 0.03, Sinkhorn blur 0.05, and evaluation over all target cells. The framework was implemented in PyTorch 2.5.1 and run on an NVIDIA RTX 4090 GPU. The dataset- and task-specific hyperparameters used in the main benchmark runs are summarized in Table S3.

Preprocessing and leakage control. For each dataset-task split, target time points listed in Table S2 were excluded from model fitting. Following scMix, library-size normalization and log-transformation were applied at the cell level and therefore did not use labels or statistics from target time points. Procedures that require fitting population-level statistics, including highly variable gene (HVG) selection, PCA for cluster-representative cell selection, feature scaling and KMeans clustering, were fitted on reference snapshots only within each split and then applied to the corresponding target snapshots for evaluation. Accordingly, the phrase "all cells" in Algorithm S1 refers to all cells from the available reference snapshots, not cells from held-out target snapshots. UMAP coordinates used for visualization were fitted only for post hoc visualization and were not used by scChronos or any baseline during training.

Benchmark fairness. All methods were evaluated on the same train-target split, the same HVG panel size, and the same target time points. For each dataset-task-HVG setting, scChronos and all baselines were compared against the same held-out target snapshots using the same OT-distance evaluation code. Unless a baseline method imposed a method-specific output size, predicted cell populations were sampled or generated to match the evaluation protocol used for scChronos, and all OT distances were computed against the full available target-cell population. Each benchmark result was summarized over five independent runs with different random seeds, and the supplementary benchmark tables report mean $\pm$ standard deviation. Baseline hyperparameters followed the recommended settings from the original implementations or papers.

Biological program evaluation. Marker and pathway analyses were used as retrospective biological assessments of predicted E19 target snapshots in the ECED Kidney forecasting task, rather than as independent training signals or model-selection criteria. The random-forest classifier used to assign pseudo cell-type labels to predicted cells was trained only after prediction, using ground-truth E19 expression profiles and metadata labels, and was used solely for cell-type-aware evaluation. Marker genes were defined from the ground-truth E19 snapshot by retaining the top 50 genes for each of the endothelial, epithelial and mesenchymal cell types after restricting to the HVG1000 panel, resulting in 150 cell-type-marker pairs for the marker-gene scatter analysis. Matched marker-program errors were then computed after aggregating marker scores within the three terminal cell types. Reactome pathway analysis used ssGSEA scores computed from the same HVG1000 expression space.

Gene-token input length. The HVG setting and the maximum gene-token length represent two different quantities. HVG500, HVG1000 and HVG2000 specify the dataset-level feature panel used to construct the expression matrix. In contrast, "Max gene tokens per cell" specifies the maximum number of nonzero expressed genes retained for an individual cell before transcriptomic encoding. When the number of nonzero genes exceeds this

value, genes with the highest expression values are retained; otherwise, all nonzero genes are used and the remaining token positions are padded.

Table S3: scChronos hyperparameters used for the main benchmark runs.

Dataset	Task	Max gene tokens per cell	Batch size	LR
Schiebinger2019	Recovery	273	128	3e-4
Schiebinger2019	Forecasting	296	128	3e-4
ECED Kidney	Recovery	416	128	3e-4
ECED Kidney	Forecasting	459	128	3e-4
Veres2019	Recovery	256	128	3e-4
Veres2019	Forecasting	256	128	3e-4
Cao2020	Recovery	256	128	3e-4
Cao2020	Forecasting	256	128	3e-4

S4 Ablation Analysis

Table S4 reports the ablation results on ECED Kidney recovery under the HVG1000 setting. For the "w/o cluster-representative cells" variant, the cluster-representative cells were replaced by the same number of randomly sampled cells from each reference snapshot. For "w/o local fusion branch", the local context embedding was removed and prediction used the global context embedding alone. Conversely, for "w/o global fusion branch", the global context embedding was removed and prediction used the local context embedding alone. All remaining model components and training settings were unchanged.

Table S4: ECED Kidney recovery ablation analysis. Values are target-time OT distances; lower is better.

Variant	t5	t7	t9	t11	t15	t16	t17	t18	Mean
**scChronos**	**34.01**	**31.01**	**33.52**	**30.81**	**38.01**	**50.53**	**37.57**	**51.13**	**38.32**
w/o cluster-representative cells	37.19	33.54	37.35	34.37	43.89	54.89	43.15	57.43	42.73
w/o local fusion branch	34.48	31.36	34.11	31.34	38.84	51.29	38.55	51.95	38.99
w/o global fusion branch	36.28	32.98	36.99	34.04	43.28	54.53	42.39	56.69	42.15

S5 Cluster-representative Cell Selection

For each reference time point r, scChronos compresses the normalized log-expression matrix $X_r \in \mathbb{R}^{n_r \times G}$ into a compact set of cluster-representative cells. KMeans is performed in PCA space fitted on reference snapshots to reduce high-dimensional noise, and the real cell nearest to each cluster centroid is retained for transcriptomic encoding. Algorithm S1 summarizes the selection procedure.

Algorithm S1 KMeans-based cluster-representative cell selection

Require: Normalized log-expression matrices $\{X_r\}_{r \in \mathcal{R}}$, maximum number of clusters K
Ensure: Cluster-representative cell sets $\{\mathcal{P}_r\}_{r \in \mathcal{R}}$

```
N ← Σ_{r∈R} n_r, d ← min(50, G, N − 1)
Fit PCA on all reference cells and obtain Z_r ∈ R^{n_r×d} for each r ∈ R
for each reference time point r ∈ R do
    K_r ← min(K, n_r)
    if n_r ≤ K then
        P_r ← {1, . . . , n_r}
    else
        {C_{r,1}, . . . , C_{r,K_r}} ← KMeans(Z_r, K_r)
        P_r ← ∅
        for each cluster C_{r,k} do
            p_{r,k} ← arg min_{i∈C_{r,k}} ‖Z_{r,i} − μ^{PCA}_{r,k}‖_2
            P_r ← P_r ∪ {p_{r,k}}
        end for
    end if
end for
return {P_r}_{r∈R}
```

S6 Supplementary Figures

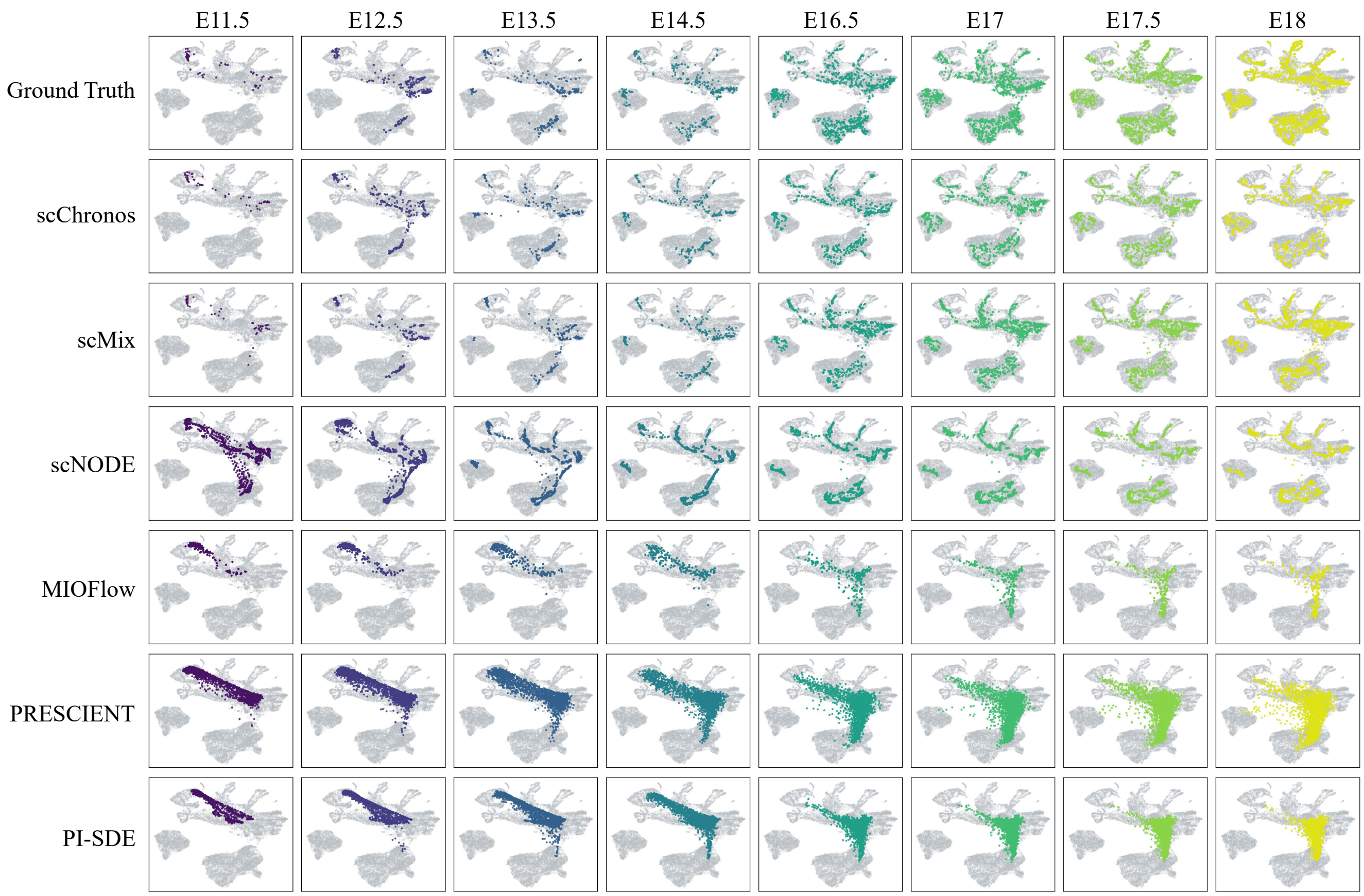

Figure S1: ECED Kidney recovery HVG1000 UMAP visualizations.

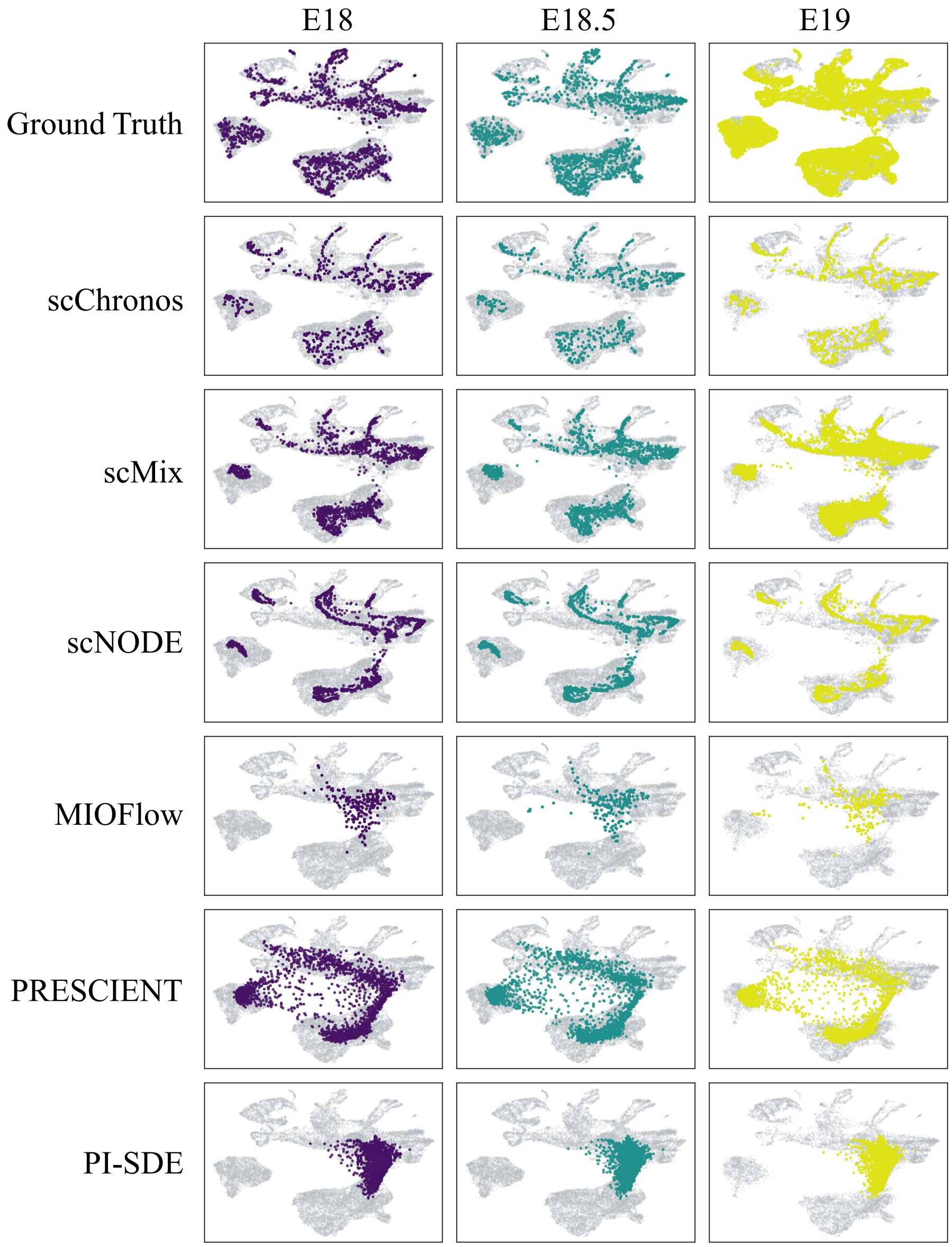

Figure S2: ECED Kidney forecasting HVG1000 UMAP visualizations.

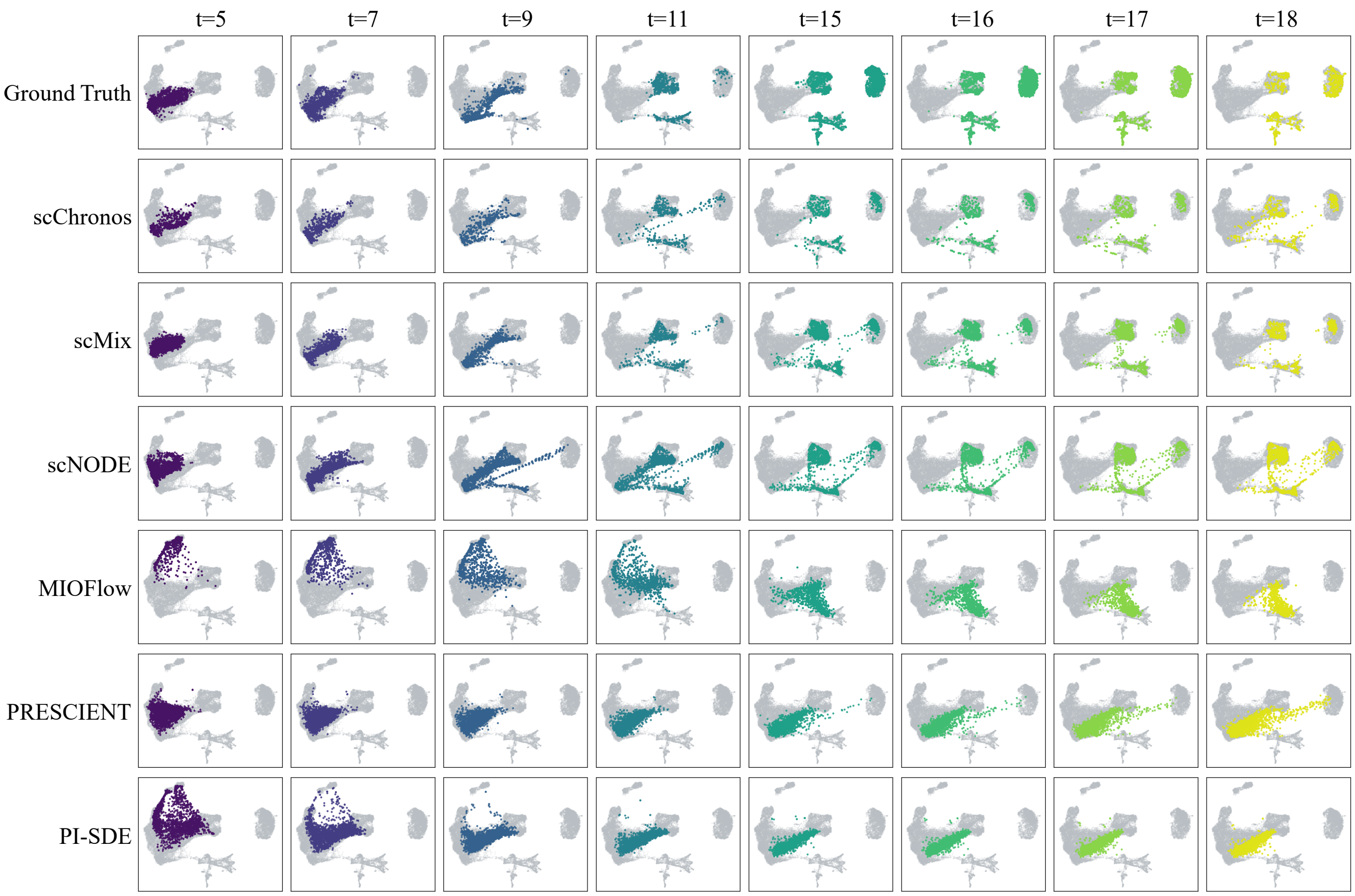

Figure S3: Schiebinger2019 recovery HVG1000 UMAP visualizations.

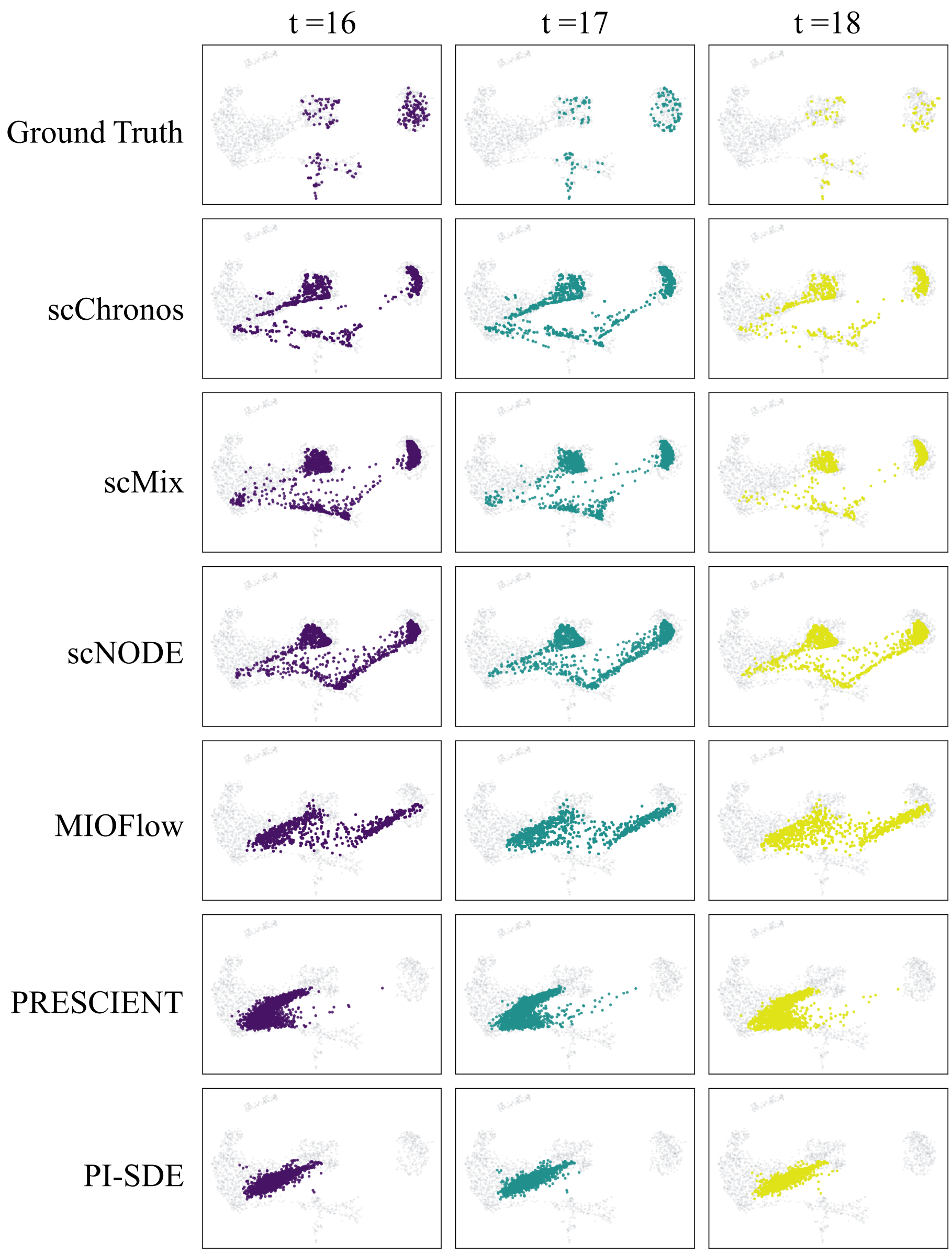

Figure S4: Schiebinger2019 forecasting HVG1000 UMAP visualizations.

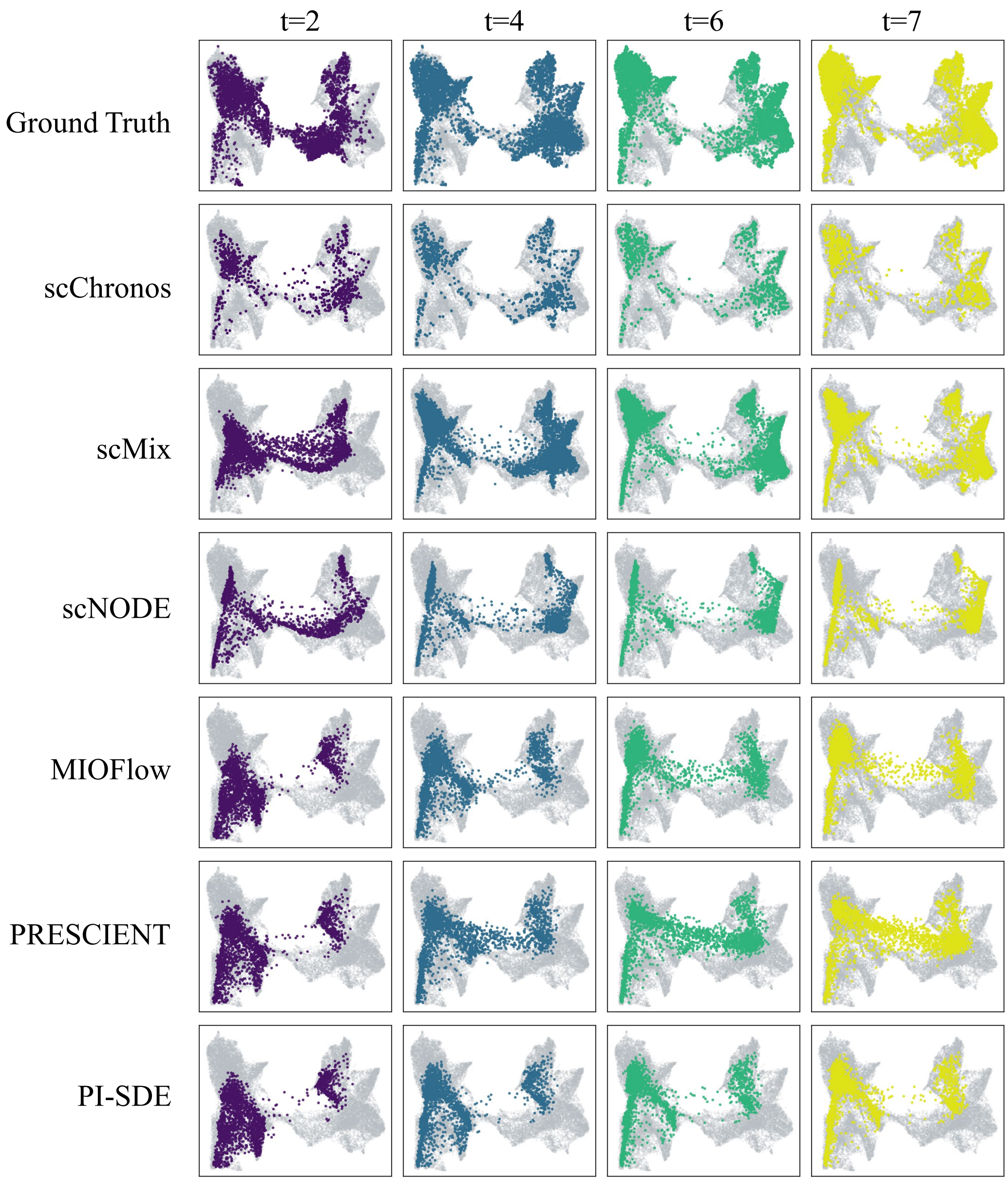

Figure S5: Veres2019 recovery HVG1000 UMAP visualizations.

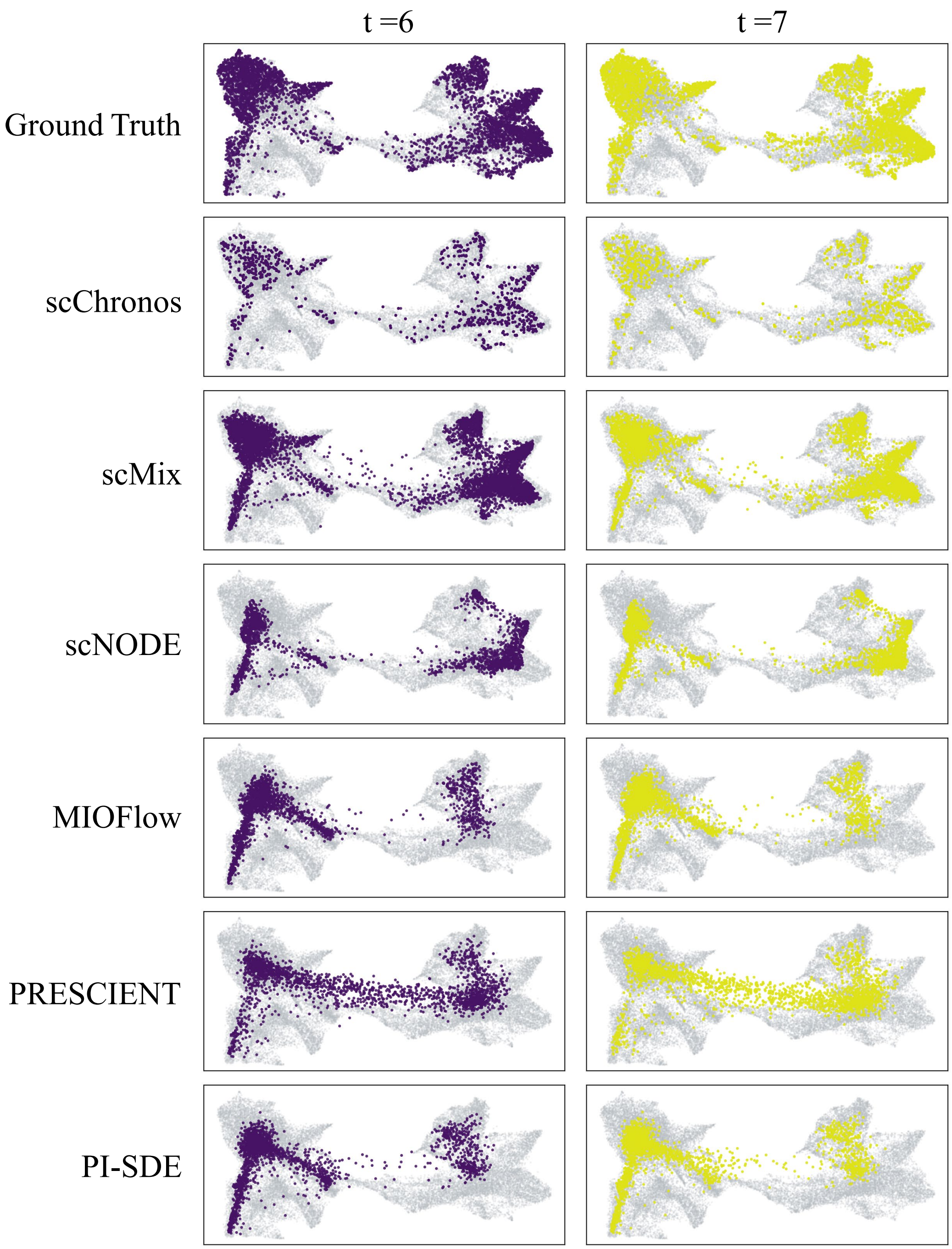

Figure S6: Veres2019 forecasting HVG1000 UMAP visualizations.

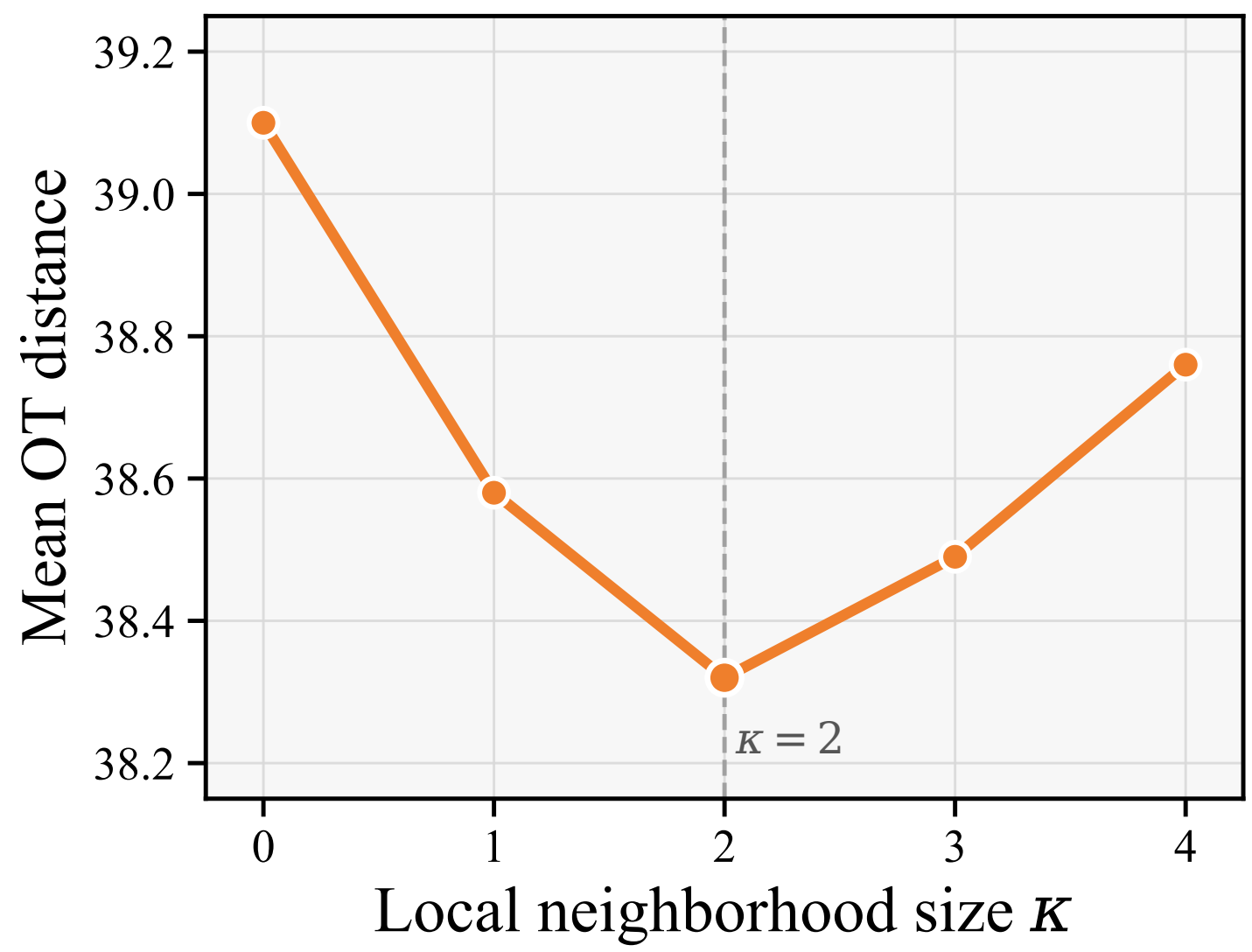

Figure S7: Parameter analysis of the local-neighborhood size κ under the setting of ECED Kidney HVG1000 recovery. Mean OT distance was evaluated under different values of κ, where lower values indicate better target-snapshot prediction. The best performance was obtained at $\kappa = 2$, which was used in the main experiments.

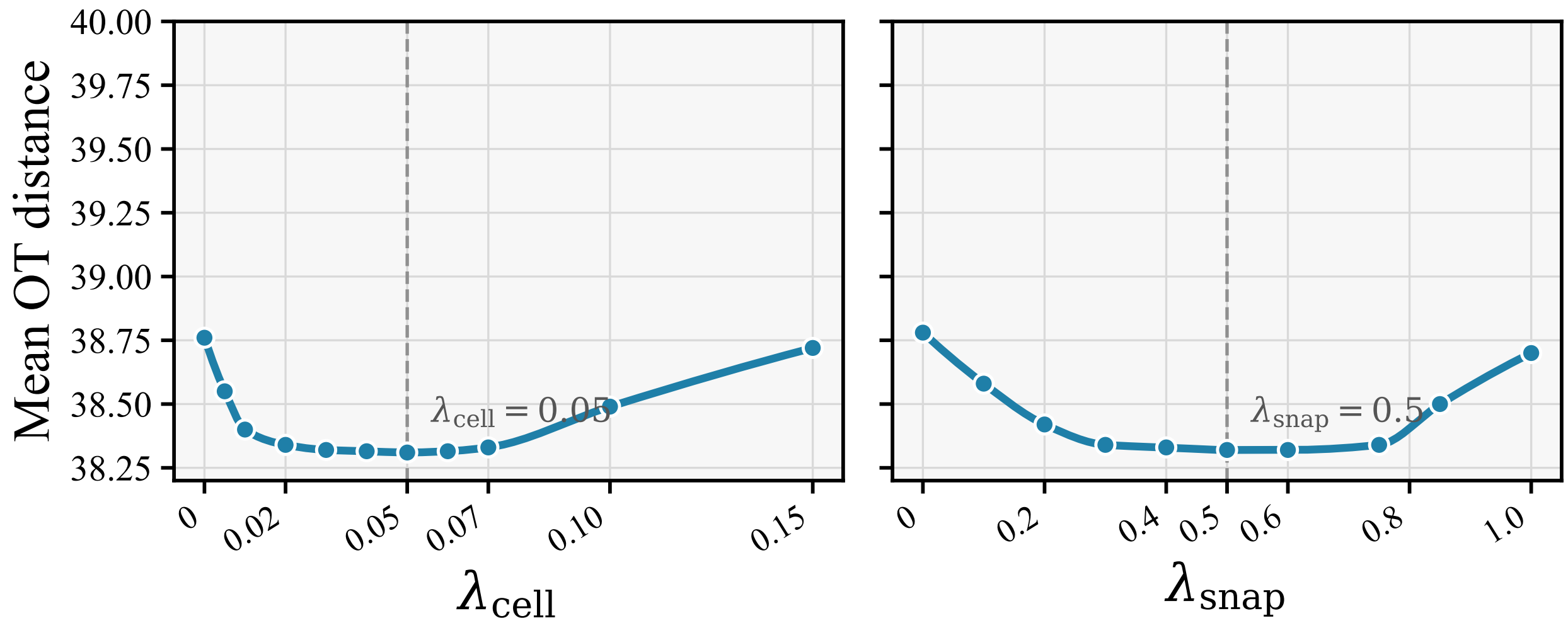

Figure S8: Parameter analysis of auxiliary loss weights under the setting of ECED Kidney HVG1000 recovery. Left: mean OT distance under different values of λ_{cell}, with the best performance obtained around $\lambda_{\text{cell}} = 0.05$. Right: mean OT distance under different values of λ_{snap}, with the best performance obtained around $\lambda_{\text{snap}} = 0.5$. The relatively flat regions around the selected values indicate that scChronos is not sensitive to small changes in the auxiliary loss weights. Lower values indicate better target-snapshot prediction.

S7 Supplementary Tables

S7.1 Recovery HVG500

Table S5: Schiebinger2019 recovery HVG500 benchmark results.

Method	t=5	t=7	t=9	t=11	t=15	t=16	t=17	t=18	Mean
scChronos	**27.96 ± 0.54**	**27.17 ± 0.65**	**46.55 ± 0.44**	**53.39 ± 0.67**	**46.62 ± 0.36**	**51.16 ± 0.42**	**43.38 ± 0.52**	**50.99 ± 0.62**	**43.40 ± 0.53**
scMix	28.36 ± 1.19	27.17 ± 1.17	47.23 ± 1.36	53.97 ± 1.43	47.36 ± 1.31	53.22 ± 1.36	46.01 ± 1.41	54.04 ± 1.36	44.67 ± 1.32
scNODE	29.26 ± 1.63	30.55 ± 1.63	48.13 ± 1.06	56.70 ± 1.15	51.28 ± 1.94	58.53 ± 1.35	51.33 ± 0.97	61.31 ± 1.28	48.39 ± 1.38
MIOFlow	75.01 ± 4.03	61.66 ± 3.22	66.42 ± 3.92	66.12 ± 2.42	69.24 ± 2.76	73.15 ± 2.93	67.58 ± 2.35	74.16 ± 3.30	69.17 ± 3.12
PRESCIENT	34.63 ± 0.90	32.10 ± 1.02	53.21 ± 1.23	80.91 ± 2.64	71.96 ± 2.18	72.63 ± 1.58	68.32 ± 1.94	73.78 ± 1.77	60.94 ± 1.66
PI-SDE	58.01 ± 1.73	45.34 ± 1.35	56.70 ± 1.68	71.17 ± 1.93	73.97 ± 2.26	76.89 ± 2.38	71.70 ± 1.90	78.29 ± 2.46	66.51 ± 1.96

Table S6: Veres2019 recovery HVG500 benchmark results.

Method	t=2	t=4	t=6	t=7	Mean
scChronos	**53.28 ± 0.83**	**52.64 ± 0.51**	**48.85 ± 0.59**	**49.63 ± 0.61**	**51.10 ± 0.63**
scMix	65.83 ± 0.97	56.42 ± 0.58	53.20 ± 0.76	54.78 ± 0.72	57.56 ± 0.76
scNODE	56.37 ± 0.80	54.16 ± 0.63	55.38 ± 0.80	56.55 ± 0.96	55.61 ± 0.80
MIOFlow	65.26 ± 2.04	62.83 ± 2.57	62.41 ± 1.87	64.13 ± 2.16	63.66 ± 2.16
PRESCIENT	63.00 ± 1.09	62.69 ± 1.36	67.77 ± 1.85	69.74 ± 1.86	65.80 ± 1.54
PI-SDE	65.21 ± 1.09	63.45 ± 1.45	64.56 ± 1.46	66.26 ± 1.49	64.87 ± 1.37

Table S7: Cao2020 recovery HVG500 benchmark results.

Method	t=2	t=4	t=6	t=7	Mean
scChronos	**4.54 ± 0.04**	**3.13 ± 0.03**	**3.43 ± 0.03**	**3.94 ± 0.03**	**3.76 ± 0.03**
scMix	9.58 ± 0.12	13.18 ± 0.25	4.49 ± 0.07	4.14 ± 0.06	7.85 ± 0.12
scNODE	7.34 ± 0.17	6.44 ± 0.16	5.67 ± 0.15	5.85 ± 0.17	6.33 ± 0.16
MIOFlow	11.98 ± 0.57	9.30 ± 0.41	8.15 ± 0.38	8.35 ± 0.39	9.45 ± 0.44
PRESCIENT	13.91 ± 0.43	12.30 ± 0.29	11.54 ± 0.34	12.15 ± 0.38	12.48 ± 0.36
PI-SDE	15.04 ± 0.50	13.34 ± 0.40	11.64 ± 0.33	11.49 ± 0.36	12.88 ± 0.40

Table S8: ECED Kidney recovery HVG500 benchmark results.

Method	t=5	t=7	t=9	t=11	t=15	t=16	t=17	t=18	Mean
scChronos	**20.47 ± 0.32**	**15.93 ± 0.31**	**20.16 ± 0.33**	**17.75 ± 0.30**	**23.42 ± 0.35**	**31.36 ± 0.36**	**22.94 ± 0.32**	**31.32 ± 0.36**	**22.92 ± 0.33**
scMix	20.73 ± 0.41	16.59 ± 0.40	22.75 ± 0.55	21.78 ± 0.46	27.00 ± 0.59	32.52 ± 0.65	27.04 ± 0.51	33.67 ± 0.65	25.26 ± 0.53
scNODE	21.98 ± 0.58	18.88 ± 0.43	21.99 ± 0.42	19.89 ± 0.39	26.36 ± 0.49	32.48 ± 0.62	25.55 ± 0.43	31.91 ± 0.56	24.88 ± 0.49
MIOFlow	30.53 ± 1.55	25.89 ± 1.69	31.24 ± 1.70	41.16 ± 2.27	46.01 ± 2.19	51.78 ± 2.64	48.99 ± 2.60	56.13 ± 3.15	41.47 ± 2.22
PRESCIENT	26.06 ± 0.72	23.24 ± 0.67	29.28 ± 0.71	35.02 ± 1.00	38.81 ± 0.99	44.56 ± 1.24	42.08 ± 0.99	49.17 ± 1.36	36.03 ± 0.96
PI-SDE	27.47 ± 0.89	24.31 ± 0.74	29.81 ± 0.86	37.20 ± 0.92	41.78 ± 1.14	48.06 ± 1.15	45.42 ± 1.13	52.56 ± 1.22	38.33 ± 1.01

S7.2 Recovery HVG1000

Table S9: Schiebinger2019 recovery HVG1000 benchmark results.

Method	t=5	t=7	t=9	t=11	t=15	t=16	t=17	t=18	Mean
scChronos	**21.31 ± 0.84**	**22.58 ± 0.74**	**41.47 ± 1.13**	**59.72 ± 0.86**	**50.05 ± 0.56**	**52.39 ± 0.75**	**47.58 ± 0.63**	**48.87 ± 0.82**	**43.00 ± 0.79**
scMix	21.48 ± 1.27	23.71 ± 1.26	46.04 ± 1.57	65.11 ± 1.60	55.22 ± 1.54	60.34 ± 1.70	56.36 ± 1.63	59.23 ± 1.57	48.44 ± 1.52
scNODE	21.98 ± 1.53	25.17 ± 1.66	43.65 ± 1.89	66.42 ± 1.46	56.48 ± 1.68	63.75 ± 1.45	58.07 ± 1.33	63.70 ± 1.58	49.90 ± 1.57
MIOFlow	60.99 ± 3.96	51.13 ± 3.17	59.33 ± 3.43	75.75 ± 3.63	73.24 ± 3.25	75.21 ± 3.48	70.93 ± 3.41	71.07 ± 3.17	67.21 ± 3.44
PRESCIENT	24.62 ± 1.74	25.92 ± 1.91	46.63 ± 1.46	83.80 ± 2.82	70.80 ± 2.14	71.15 ± 1.88	69.07 ± 1.88	66.91 ± 1.74	57.36 ± 1.95
PI-SDE	41.18 ± 1.20	34.50 ± 1.11	49.09 ± 1.64	78.84 ± 2.36	74.81 ± 2.21	76.33 ± 2.49	73.62 ± 2.35	72.07 ± 2.12	62.55 ± 1.94

Table S10: Veres2019 recovery HVG1000 benchmark results.

Method	t=2	t=4	t=6	t=7	Mean
scChronos	**94.90 ± 0.67**	**93.16 ± 0.59**	**89.33 ± 0.44**	**90.74 ± 0.71**	**92.04 ± 0.69**
scMix	110.09 ± 1.08	98.72 ± 1.03	96.24 ± 0.68	98.11 ± 0.82	100.79 ± 0.90
scNODE	101.43 ± 1.18	98.60 ± 1.27	100.24 ± 1.36	100.85 ± 1.50	100.28 ± 1.33
MIOFlow	111.84 ± 4.12	106.31 ± 3.73	104.72 ± 2.96	105.61 ± 3.35	107.12 ± 3.54
PRESCIENT	109.01 ± 1.98	109.23 ± 2.68	113.96 ± 2.78	115.21 ± 2.59	111.85 ± 2.51
PI-SDE	112.17 ± 2.63	110.83 ± 2.57	112.18 ± 2.26	113.74 ± 2.34	112.23 ± 2.45

Table S11: Cao2020 recovery HVG1000 benchmark results.

Method	t=2	t=4	t=6	t=7	Mean
scChronos	**9.30 ± 0.95**	**6.14 ± 0.75**	**6.38 ± 0.78**	**6.52 ± 0.71**	**7.09 ± 0.80**
scMix	20.09 ± 1.25	17.84 ± 1.34	8.42 ± 1.10	7.83 ± 1.08	13.54 ± 1.19
scNODE	16.08 ± 1.31	13.86 ± 1.28	12.50 ± 1.31	12.49 ± 1.32	13.73 ± 1.31
MIOFlow	19.30 ± 1.59	14.82 ± 1.51	13.69 ± 1.62	13.70 ± 1.54	15.38 ± 1.56
PRESCIENT	21.03 ± 2.05	17.26 ± 1.81	16.59 ± 1.89	16.99 ± 1.97	17.97 ± 1.93
PI-SDE	23.92 ± 2.30	20.92 ± 2.23	19.50 ± 2.25	19.21 ± 2.13	20.88 ± 2.23

Table S12: ECED Kidney recovery HVG1000 benchmark results.

Method	t=5	t=7	t=9	t=11	t=15	t=16	t=17	t=18	Mean
scChronos	**34.01 ± 0.49**	**31.01 ± 0.32**	**33.52 ± 0.46**	**30.81 ± 0.34**	**38.01 ± 0.49**	**50.53 ± 0.57**	**37.57 ± 0.53**	**51.13 ± 0.67**	**38.32 ± 0.42**
scMix	34.29 ± 0.72	31.86 ± 0.65	37.45 ± 0.75	34.41 ± 0.73	46.62 ± 0.97	55.67 ± 0.98	45.35 ± 0.96	61.28 ± 1.07	43.37 ± 0.85
scNODE	39.85 ± 1.14	32.66 ± 1.00	37.44 ± 0.96	34.15 ± 0.91	42.52 ± 1.04	54.75 ± 1.14	41.16 ± 0.97	55.34 ± 1.38	42.23 ± 1.07
MIOFlow	51.70 ± 2.76	43.98 ± 2.45	50.23 ± 2.65	61.56 ± 3.22	69.75 ± 3.34	78.55 ± 4.27	73.26 ± 4.18	84.89 ± 4.41	64.24 ± 3.41
PRESCIENT	43.91 ± 1.11	38.88 ± 0.98	48.72 ± 1.32	56.36 ± 1.30	63.52 ± 1.82	72.64 ± 1.70	68.16 ± 1.95	80.43 ± 1.94	59.08 ± 1.51
PI-SDE	47.03 ± 1.35	41.15 ± 1.12	49.91 ± 1.31	59.46 ± 1.65	67.50 ± 2.05	77.69 ± 2.02	73.24 ± 2.00	87.18 ± 2.75	62.89 ± 1.78

S7.3 Recovery HVG2000

Table S13: Schiebinger2019 recovery HVG2000 benchmark results.

Method	t=5	t=7	t=9	t=11	t=15	t=16	t=17	t=18	Mean
scChronos	**49.41 ± 0.82**	**51.52 ± 0.77**	**94.94 ± 1.54**	**128.90 ± 2.38**	**103.47 ± 0.88**	**111.25 ± 1.04**	**102.13 ± 0.55**	**108.12 ± 0.78**	**93.72 ± 1.09**
scMix	51.26 ± 1.06	52.61 ± 0.55	102.95 ± 1.96	129.30 ± 3.05	104.55 ± 0.98	115.03 ± 1.41	103.50 ± 0.70	110.60 ± 1.00	96.23 ± 1.34
scNODE	54.51 ± 0.69	59.03 ± 2.84	103.41 ± 2.55	142.84 ± 5.06	131.79 ± 8.07	147.25 ± 10.05	103.87 ± 8.64	110.31 ± 8.27	106.63 ± 5.77
MIOFlow	53.99 ± 1.42	64.99 ± 2.75	110.27 ± 2.73	158.06 ± 10.09	171.08 ± 17.12	213.34 ± 41.84	222.37 ± 66.47	264.19 ± 104.06	157.29 ± 30.81
PRESCIENT	88.89 ± 0.64	90.76 ± 0.90	116.69 ± 0.81	141.21 ± 0.43	154.10 ± 2.32	165.02 ± 2.69	150.26 ± 2.37	158.22 ± 2.48	133.14 ± 1.58
PI-SDE	119.30 ± 1.09	142.51 ± 1.62	201.46 ± 1.46	258.20 ± 2.25	298.50 ± 4.34	332.24 ± 5.80	332.66 ± 5.44	365.60 ± 6.99	256.31 ± 3.62

Table S14: Veres2019 recovery HVG2000 benchmark results.

Method	t=2	t=4	t=6	t=7	Mean
scChronos	**177.55 ± 0.69**	**175.97 ± 0.72**	**169.47 ± 0.67**	**169.40 ± 0.64**	**173.10 ± 0.68**
scMix	190.16 ± 1.62	178.72 ± 1.28	175.00 ± 1.25	176.20 ± 1.24	180.02 ± 1.35
scNODE	182.51 ± 2.60	181.45 ± 3.05	181.97 ± 2.72	180.26 ± 3.12	181.55 ± 2.87
MIOFlow	198.60 ± 7.31	193.58 ± 6.46	189.69 ± 5.62	187.47 ± 6.61	192.33 ± 6.50
PRESCIENT	195.88 ± 3.55	197.29 ± 4.60	200.61 ± 4.55	199.71 ± 5.43	198.37 ± 4.53
PI-SDE	199.27 ± 4.35	199.16 ± 4.91	199.15 ± 3.79	198.85 ± 3.92	199.11 ± 4.24

Table S15: Cao2020 recovery HVG2000 benchmark results.

Method	t=2	t=4	t=6	t=7	Mean
scChronos	**20.22 ± 0.23**	**14.63 ± 0.44**	**16.80 ± 0.09**	**16.35 ± 0.11**	**17.00 ± 0.22**
scMix	37.97 ± 0.66	25.04 ± 0.22	18.88 ± 0.18	16.71 ± 0.16	24.65 ± 0.30
scNODE	32.43 ± 0.37	24.90 ± 0.34	24.13 ± 0.47	22.87 ± 0.50	26.09 ± 0.42
MIOFlow	33.63 ± 0.87	25.82 ± 0.80	25.15 ± 1.00	23.74 ± 0.92	27.09 ± 0.90
PRESCIENT	35.00 ± 0.79	28.50 ± 0.65	28.60 ± 0.92	27.93 ± 0.75	30.00 ± 0.78
PI-SDE	37.82 ± 1.08	32.16 ± 0.88	31.60 ± 0.89	30.34 ± 0.90	32.98 ± 0.94

Table S16: ECED Kidney recovery HVG2000 benchmark results.

Method	t=5	t=7	t=9	t=11	t=15	t=16	t=17	t=18	Mean
scChronos	**55.88 ± 0.48**	**48.40 ± 0.41**	**56.44 ± 0.47**	**51.44 ± 0.35**	**65.27 ± 0.39**	**83.49 ± 0.50**	**63.97 ± 0.37**	**86.17 ± 0.43**	**63.88 ± 0.43**
scMix	56.70 ± 0.46	49.30 ± 0.34	57.25 ± 0.45	52.40 ± 0.55	68.50 ± 0.95	85.16 ± 0.92	66.62 ± 0.69	88.34 ± 1.09	65.53 ± 0.68
scNODE	58.76 ± 1.53	51.93 ± 1.18	61.58 ± 1.44	55.79 ± 1.44	68.39 ± 1.18	85.98 ± 2.08	67.48 ± 1.53	87.72 ± 1.40	67.20 ± 1.47
MIOFlow	73.11 ± 4.32	63.69 ± 4.13	75.46 ± 3.74	88.01 ± 4.42	101.68 ± 6.77	117.61 ± 6.66	108.50 ± 5.42	128.59 ± 5.86	94.58 ± 5.17
PRESCIENT	66.62 ± 2.18	58.63 ± 1.76	73.65 ± 2.32	82.20 ± 2.15	92.22 ± 2.89	108.84 ± 2.78	98.37 ± 3.01	120.74 ± 3.67	87.66 ± 2.59
PI-SDE	70.25 ± 2.40	62.65 ± 1.79	77.25 ± 2.47	88.19 ± 3.06	99.94 ± 2.51	116.60 ± 2.92	107.19 ± 2.73	129.99 ± 4.17	94.01 ± 2.76

S7.4 Forecasting HVG500

Table S17: Schiebinger2019 forecasting HVG500 benchmark results.

Method	t=16	t=17	t=18	Mean
scChronos	**43.09 ± 0.74**	**36.78 ± 0.82**	**43.00 ± 0.41**	**40.96 ± 0.66**
scMix	46.43 ± 0.89	41.87 ± 0.96	49.55 ± 0.65	45.95 ± 0.83
scNODE	48.61 ± 1.06	45.28 ± 1.19	55.14 ± 1.23	49.68 ± 1.16
MIOFlow	69.45 ± 3.13	62.09 ± 3.54	68.05 ± 3.91	66.53 ± 3.53
PRESCIENT	73.00 ± 2.52	67.61 ± 2.71	73.66 ± 2.83	71.43 ± 2.69
PI-SDE	72.62 ± 1.93	67.05 ± 2.05	73.46 ± 2.29	71.04 ± 2.09

Table S18: Veres2019 forecasting HVG500 benchmark results.

Method	t=6	t=7	Mean
scChronos	**47.13 ± 0.43**	**47.30 ± 0.51**	**47.22 ± 0.47**
scMix	55.22 ± 0.74	56.24 ± 0.59	55.73 ± 0.67
scNODE	55.21 ± 0.86	55.87 ± 0.72	55.54 ± 0.79
MIOFlow	62.27 ± 2.37	63.27 ± 2.96	62.77 ± 2.67
PRESCIENT	63.32 ± 1.82	65.52 ± 1.84	64.42 ± 1.83
PI-SDE	64.45 ± 1.78	66.05 ± 1.63	65.25 ± 1.71

Table S19: Cao2020 forecasting HVG500 benchmark results.

Method	t=6	t=7	Mean
scChronos	**3.42 ± 0.17**	**4.94 ± 0.19**	**4.18 ± 0.18**
scMix	4.26 ± 0.26	5.97 ± 0.29	5.12 ± 0.28
scNODE	7.12 ± 0.26	8.83 ± 0.27	7.98 ± 0.27
MIOFlow	9.42 ± 0.61	10.64 ± 0.62	10.03 ± 0.61
PRESCIENT	6.10 ± 0.29	7.14 ± 0.20	6.62 ± 0.24
PI-SDE	12.97 ± 0.46	13.59 ± 0.51	13.28 ± 0.48

Table S20: ECED Kidney forecasting HVG500 benchmark results.

Method	t=18	t=19	t=20	Mean
scChronos	**32.67 ± 0.52**	**28.45 ± 0.69**	**38.05 ± 0.76**	**33.06 ± 0.66**
scMix	41.85 ± 0.82	34.92 ± 0.49	46.20 ± 0.73	40.99 ± 0.68
scNODE	41.94 ± 1.06	37.20 ± 0.84	48.34 ± 1.02	42.49 ± 0.97
MIOFlow	65.33 ± 3.72	63.22 ± 4.18	74.27 ± 4.76	67.61 ± 4.22
PRESCIENT	61.67 ± 1.49	58.33 ± 1.81	70.47 ± 2.07	63.49 ± 1.79
PI-SDE	65.86 ± 2.41	62.60 ± 1.98	73.57 ± 1.82	67.35 ± 2.07

S7.5 Forecasting HVG1000

Table S21: Schiebinger2019 forecasting HVG1000 benchmark results.

Method	t=16	t=17	t=18	Mean
scChronos	**51.64 ± 0.94**	**47.57 ± 0.97**	**47.45 ± 1.12**	**48.89 ± 1.01**
scMix	56.83 ± 1.10	53.76 ± 0.89	55.74 ± 1.06	55.44 ± 1.02
scNODE	63.45 ± 1.57	58.18 ± 1.28	59.69 ± 1.43	60.44 ± 1.43
MIOFlow	78.90 ± 4.43	73.87 ± 5.07	73.43 ± 4.83	75.40 ± 4.78
PRESCIENT	78.17 ± 2.66	73.66 ± 2.55	72.06 ± 2.00	74.63 ± 2.40
PI-SDE	81.67 ± 3.36	79.42 ± 2.62	77.75 ± 3.24	79.62 ± 3.07

Table S22: Veres2019 forecasting HVG1000 benchmark results.

Method	t=6	t=7	Mean
scChronos	**87.23 ± 0.44**	**86.93 ± 0.63**	**87.08 ± 0.54**
scMix	101.76 ± 1.20	102.62 ± 1.06	102.19 ± 1.13
scNODE	96.98 ± 1.34	97.58 ± 1.30	97.28 ± 1.32
MIOFlow	105.93 ± 3.47	106.84 ± 3.58	106.38 ± 3.53
PRESCIENT	107.90 ± 2.98	110.08 ± 2.59	108.99 ± 2.79
PI-SDE	109.05 ± 2.60	110.74 ± 2.73	109.89 ± 2.67

Table S23: Cao2020 forecasting HVG1000 benchmark results.

Method	t=6	t=7	Mean
scChronos	**8.21 ± 0.37**	**10.71 ± 0.26**	**9.46 ± 0.32**
scMix	9.50 ± 0.41	11.55 ± 0.36	10.52 ± 0.39
scNODE	13.91 ± 0.45	16.32 ± 0.48	15.12 ± 0.46
MIOFlow	15.89 ± 0.85	17.39 ± 1.00	16.64 ± 0.93
PRESCIENT	12.00 ± 0.49	13.55 ± 0.69	12.78 ± 0.59
PI-SDE	19.24 ± 0.72	20.28 ± 0.62	19.76 ± 0.67

Table S24: ECED Kidney forecasting HVG1000 benchmark results.

Method	t=18	t=19	t=20	Mean
scChronos	**59.56 ± 0.98**	**48.32 ± 1.21**	**62.42 ± 0.74**	**56.77 ± 0.98**
scMix	67.82 ± 1.22	56.48 ± 1.09	74.56 ± 0.94	66.29 ± 1.08
scNODE	73.07 ± 1.64	65.80 ± 1.52	83.15 ± 2.21	74.01 ± 1.79
MIOFlow	99.75 ± 4.97	92.05 ± 5.27	110.14 ± 5.25	100.65 ± 5.16
PRESCIENT	90.84 ± 2.84	83.49 ± 2.18	98.49 ± 2.99	90.94 ± 2.67
PI-SDE	103.61 ± 3.18	94.40 ± 3.46	112.51 ± 4.15	103.51 ± 3.60

S7.6 Forecasting HVG2000

Table S25: Schiebinger2019 forecasting HVG2000 benchmark results.

Method	t=16	t=17	t=18	Mean
scChronos	**108.09 ± 0.74**	**98.03 ± 1.21**	**100.66 ± 0.84**	**102.26 ± 0.93**
scMix	114.90 ± 0.79	106.54 ± 1.36	111.79 ± 1.19	111.08 ± 1.11
scNODE	173.89 ± 7.01	157.73 ± 5.62	178.87 ± 4.40	170.16 ± 5.68
MIOFlow	204.41 ± 13.30	183.09 ± 19.97	197.81 ± 37.79	195.11 ± 23.69
PRESCIENT	210.16 ± 7.28	187.23 ± 6.78	203.70 ± 6.90	200.36 ± 6.99
PI-SDE	218.88 ± 4.36	200.51 ± 4.19	214.30 ± 5.25	211.23 ± 4.60

Table S26: Veres2019 forecasting HVG2000 benchmark results.

Method	t=6	t=7	Mean
scChronos	**167.59 ± 0.82**	**164.91 ± 0.76**	**166.25 ± 0.79**
scMix	188.09 ± 1.92	188.16 ± 2.30	188.13 ± 2.11
scNODE	187.07 ± 3.06	185.16 ± 2.64	186.12 ± 2.85
MIOFlow	190.93 ± 7.83	189.79 ± 6.75	190.36 ± 7.29
PRESCIENT	192.14 ± 5.38	191.82 ± 4.93	191.98 ± 5.15
PI-SDE	198.99 ± 4.77	199.28 ± 6.18	199.13 ± 5.47

Table S27: Cao2020 forecasting HVG2000 benchmark results.

Method	t=6	t=7	Mean
scChronos	**20.62 ± 0.33**	**23.25 ± 0.44**	**21.93 ± 0.39**
scMix	21.39 ± 0.75	23.48 ± 0.69	22.44 ± 0.72
scNODE	28.22 ± 0.81	31.06 ± 0.83	29.64 ± 0.82
MIOFlow	28.42 ± 1.30	29.58 ± 1.44	29.00 ± 1.37
PRESCIENT	25.52 ± 0.58	27.30 ± 0.65	26.41 ± 0.61
PI-SDE	34.02 ± 1.08	35.26 ± 1.08	34.64 ± 1.08

Table S28: ECED Kidney forecasting HVG2000 benchmark results.

Method	t=18	t=19	t=20	Mean
scChronos	**86.97 ± 0.60**	**72.13 ± 0.51**	**93.71 ± 0.60**	**84.27 ± 0.57**
scMix	106.01 ± 1.26	89.50 ± 1.04	114.53 ± 1.35	103.35 ± 1.22
scNODE	107.09 ± 2.80	93.92 ± 2.28	119.76 ± 3.07	106.92 ± 2.72
MIOFlow	141.05 ± 9.23	129.68 ± 8.26	152.14 ± 8.47	140.96 ± 8.65
PRESCIENT	134.63 ± 4.52	119.85 ± 3.61	143.17 ± 4.21	132.55 ± 4.11
PI-SDE	153.33 ± 5.20	140.63 ± 6.00	164.62 ± 6.03	152.86 ± 5.74
